# Sex-Linked Loci on the W Chromosome in the Multi-Ocellated Racerunner (*Eremias multiocellata*) Confirm Genetic Sex-Determination Stability in Lacertid Lizards

**DOI:** 10.3390/ani13132180

**Published:** 2023-07-03

**Authors:** Zhangqing Chu, Ziwen Wang, Yuchi Zheng, Yun Xia, Xianguang Guo

**Affiliations:** 1 Chengdu Institute of Biology, Chinese Academy of Sciences, Chengdu 610041, China; chuzq@cib.ac.cn (Z.C.); wangzw@cib.ac.cn (Z.W.); zhengyc@cib.ac.cn (Y.Z.); 2 University of Chinese Academy of Sciences, Beijing 100049, China

**Keywords:** GBS, sex chromosomes, lacertid, homology, conservation

## Abstract

**Simple Summary:**

The sex-determination pattern of reptiles is the most diverse among the amniotes. At present, there is still controversy about the sex-determination mechanism of *Eremias multiocellata*. We carried out genotyping by sequencing and bioinformatics analysis on *E. multiocellata*. The results show that it has genetic sex determination (GSD) under female heterogamety, which also shows that the sex chromosome is generally conservative in the lacertid lizards.

**Abstract:**

The multi-ocellated racerunner, *Eremias multiocellata*, was considered to have temperature-dependent sex determination (TSD), as its sex ratio can be influenced at different temperatures. However, such an observation contrasts with recent findings that suggest TSD is less common than previously thought. Here, a genotyping-by-sequencing (GBS) approach was employed to identify sex-linked markers in the *E. multiocellata*, for which the mechanism choice of TSD or GSD is still controversial. We preliminarily identified 119 sex-linked markers based on sex-associated sex-specific sequences, 97% of which indicated female heterogamety. After eliminating the false positives, 38 sex-linked markers were recognized, all of which showed the ZW/ZZ system. Then, eight of the novel markers were verified by PCR amplification from 15 populations of *E. multiocellata*, which support the GSD in *E. multiocellata* without geographic variation. To test the conservation of sex chromosome in *Eremias*, the eight markers were further cross-tested by PCR amplification in 10 individuals of the Mongolian racerunner (*Eremias argus*), two of which exhibited cross-utility. The novel sex-linked markers could be mapped on the W chromosome of the sand lizard (*Lacerta agilis*). Our finding that the sex-linked markers are shared in closely related species, along with a conserved synteny of the W chromosome, further supports the homology and conservation of sex chromosomes in the lacertid lizards.

## 1. Introduction

Unlike birds and mammals, reptiles have the most diverse sex chromosomes, sex determination, and reproductive patterns among the amniotes. For reptiles, sex determination mechanisms can generally be divided into two types, namely genetic sex determination (GSD) under either XY or ZW, and temperature-dependent sex determination (TSD) [1]. Both GSD and TSD have been described in reptiles, and there is evidence that evolutionary transitions between the two modes have frequently occurred [2,3,4]. Among reptiles, lizards exhibit an extraordinary diversity of sex chromosome systems, from homomorphic to heteromorphic and completely heterochromatic sex chromosomes [5,6].

Recently, the TSD classification has been challenged by newly isolated genetic sex-linked markers, which indicate that either GSD or TSD sex determination is not a dichotomous trait [7]. Previous studies found that temperature could affect the sex ratio of squamate reptiles, suggesting that their sex determination is TSD, but recent studies indicate that these squamates have GSD, which could be influenced by temperature (temperature-influenced GSD) [8,9]. For example, the sex of the Australian bearded dragon lizard (*Pogona vitticeps*) is determined by the interaction of genes on its sex chromosomes with its incubation environment [10,11,12]. Temperature-influenced sex determination has been demonstrated in Australian agamids, and they show both TSD and female heterogamety (ZW) [12,13]. Moreover, most species in the family Lacertidae possess female heterogamety, while some of them are TSD and ZW co-occurring [14,15]. Interestingly, the spotted snow skink (*Niveoscinus ocellatus*) was found to have different sex determination mechanisms in different populations [9]. In a highland population of *N. ocellatus*, sex is genetically determined, whereas in a lowland population, the sex ratio of offspring is influenced by temperature [9]. Temperature-influenced sex determination was demonstrated in the collared lizard (*Crotaphytus collaris*) by qPCR, and extreme incubation temperature will influence its hatchling sex ratio [7]. Thus, this lizard is thought to be the first non-Australian lizard species exhibiting a temperature override of genotypic sex determination. Such newly discovered GSD in the TSD squamates can be attributed to the sex-determination mechanism that can transition from TSD to GSD, driven by the natural selection [16], but also can be explained by erroneous identified “TSD” species, which caused an overestimation of the number of GSD-to-TSD transitions among amniotes and undermined the long-term stability of GSD systems [17].

In the Lacertidae family, female heterozygosity exists in approximately 20% of lacertids [17]. In combination with qPCR of several sex-linked genes, ZW sex chromosomes were shown in 16 lacertid species [18,19], which implied little evidence for switches to TSD and turnover of sex chromosomes in lacertid lizards [17,18]. However, some species have been proposed as TSD, which challenged the long-term stability of the divergent ZZ/ZW sex chromosomes in Lacertidae [17]. *Eremias multiocellata*, the multi-ocellated racerunner, is a viviparous lizard distributed in north China, Mongolia, and the Tuva Republic of Russia [20]. The karyotype of *E. multiocellata* was 2n = 36I + 2m or 2n = 34I + 4m, with no sign of heteromorphic sex chromosomes [21,22]. A previous study indicated that *E. multiocellata* was TSD, as demonstrated by incubation experiments influencing sex ratio at different temperatures [8,23]. Afterwards, a ZW/ZZ sex-determination system was observed in *E. multiocellata* by comparative genomic hybridization (CGH) [22], and the authors also found that the W and Z chromosomes are homozygous using high-resolution cytogenetic methods. Accordingly, both TSD and GSD were proposed in *E. multiocellata*. Nevertheless, the incubation experiments lacked two critical steps—they did not exclude differential mortality between the sexes at a particular temperature and did not consider temperature-mediated sexual reversal [17]. Therefore, the focus of the debate is regarding whether it is a purely TSD (sex determination determined only by temperature) or just temperature-influenced GSD. This study will examine the sex chromosomes of *E. multiocellata* and try to clarify its sex-determining mode. 

Many studies have been conducted to determine the homology of lacertid sex chromosomes via the Z chromosome but lacking the information from the W chromosome. For example, by transcriptome sequencing and qPCR, it was found that the genomic coverage of many genes (e.g., *btk*, *dock11*, *enox2*, *f8*, *faah2*, *klhl13*, *LOC100559614*, *lpar4*, etc.) in the female Asian grass lizards (*Takydromus sexlineatus*) was half that in males, indicating Z-linkage [24]. The sex chromosomes of lizards, snakes, and birds are not homologous, but presumably by coincidence, these *T. sexlineatus* Z-genes have homologs on the human X chromosome. Meanwhile, most of their results were based on cytogenetic observations, and part of the study was based on qPCR [17,18]. On the one hand, the cytogenetic data came from karyotypes and chromosome smears, which were not accurate enough to target the detailed regions of sex-linkage. On the other hand, according to the original qPCR study, it is currently unknown which genes and how large a range of chromosomes are involved in sex linkage (targeting the genes on Z chromosome). Comparatively, the multiple sex-linked alleles on both Z and W chromosomes could not only test the homology and conservation of the sex-determination system and sex chromosomes but also decipher the recombination suppression of sex chromosome differentiation. Comparing the complete set of Z-linked genes with the W chromosome’s gene content and the W gene sequences can be used to infer the extent of non-recombining versus recombining regions [25,26]. As such, it is necessary to verify the homology of sex chromosomes of lacertids through the W chromosome. 

Genotyping by sequencing (GBS) is one of the most widely used, simplified genomic genotyping methods in non-model organisms, especially to identify the female- or male-specific markers on the W/Y chromosome [27,28]. In recent years, GBS has been brilliant in the field of identifying sex-specific markers. The GBS approach has been applied successfully in the detection of sex markers in more than 20 ranid species and several other amphibians [29,30,31]. In reptiles, GBS has been mostly applied in a variety of fields, including phylogenetic analysis, construction of evolutionary models, inference of genetic differentiation distances, and so on [32,33]. For instance, detecting differences in sex-related markers of *N. ocellatus* by GBS [9], each population appeared to have shared loci and a similar number of sex-linked loci as well as male heterogamety (XY). 

Here, we collected 48 individuals from different geographic populations to identify the sex-determination mechanism (TSD or GSD) and the sex-determination system (XY or ZW) in *E. multiocellata*. Using GBS analysis, we found that *E. multiocellata* possesses a ZZ/ZW sex chromosome system with GSD. Moreover, our results support a temperature-influenced sex-determination system in *E. multiocellata* and that the W chromosomes are conserved in Lacertidae. These findings will provide novel insight into the sex-determination mechanism of *E. multiocellata* and facilitate studies on gonad development and sex control in this lizard.

## 2. Materials and Methods

### 2.1. Sampling and Preparation

**Figure 1:**
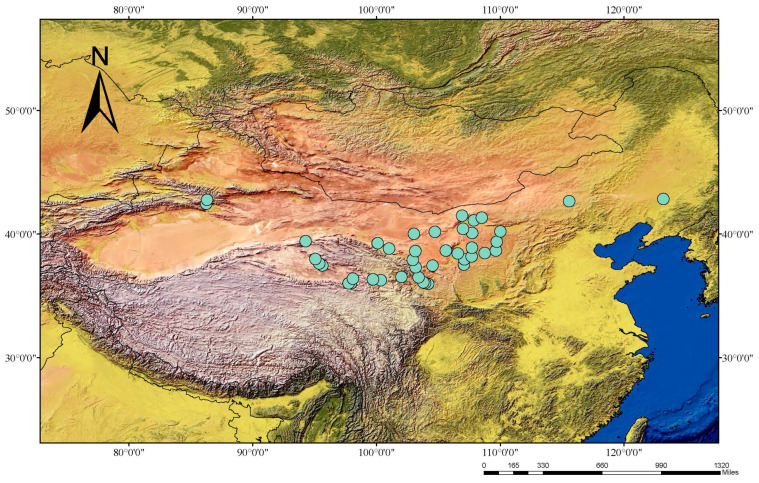
Sampling sites for the specimens of *Eremias multiocellata*. (The blue circle indicates the location of the sampling point of *E. multiocellata*).

In total, 48 adults of *E. multiocellata* (17 females and 31 males) were captured alive by hand from Ningxia, Shannxi, Inner Mongolia, Xinjiang, Qinghai, and Gansu, China (one or two individuals per population) (Figure 1; Supplementary Table S1). Physiological sex was determined by hemipenis eversion, which has been confirmed to accurately establish sexual identity in repeatability trials in a variety of reptilian taxa [34]. Animals were euthanized with an overdose of sodium pentobarbital via intraperitoneal injection, and liver tissues were extracted and preserved in 95% ethanol following the animal-use protocols approved by Chengdu Institute of Biology (CIB), Chinese Academy of Sciences. When an adult is euthanized, sex can be further confirmed by dissection of gonads. The 48 samples were used for GBS, and we selected 20 samples (10 males and 10 females) for analysis, while an additional 22 samples were used for PCR verification (Supplementary Table S1). All the voucher specimens of lizards were deposited in CIB.

### 2.2. DNA Extraction and Genotyping by Sequencing

Total genomic DNA was extracted from muscular tissues for each sample using the Qiagen^®^ DNeasy Blood and Tissue extraction kit (QIAGEN, Valencia, CA, USA) according to the manufacturer’s protocol. Following the protocol of Elshire et al. [35], we generated the GBS libraries. First, genomic DNA was digested using MseI restriction enzymes (New England Biolabs, NEB, Ipswich, MA, USA) at 37 °C. Next, after adding a sequencing junction at the end of the enzyme digestion, the purified samples were subjected to PCR reactions using a Gel Extraction Kit (QIAGEN, Valencia, CA, USA), and fragments retaining approximately 300–350 bp were isolated from agarose gels. Moreover, using a 150 bp paired-end protocol, the obtained library was sequenced on the Illumina NovaSeq6000 platform at Novogene Bioinformatics Technology Co., Ltd., Beijing, China (www.novogene.cn; accessed on 15 June 2022). Finally, the original sequences (sequencing reads) are obtained, which we call raw data. 

### 2.3. Filtering and SNP Calling

We obtained GBS clean data using software package Stacks v-2.55 [36]. Firstly, to remove the reads of low quality (e.g., reads missing the restriction site), we used the *process_radtag* algorithm in the Stacks v-2.55 program to filter the raw GBS data. Secondly, we ran the Stacks program *de novo_map*. Notably, there are three main parameters in the program, namely minimum depth of coverage (m), maximum number of mismatches between stacks (alleles) within an individual (M), and maximum number of mismatches between individuals (n). Finally, we tried to determine the best combination of parameters and applied it to the entire dataset.

### 2.4. Screening Sex-Linked Markers

Sex-linked markers are located on the heterogametic sex chromosome: the Y chromosome markers in male-heterogametic species and W chromosome markers in female-heterogametic species. It is possible to determine whether a species has a male-heterogametic sex-determination system (XY) or a female-heterogametic sex-determination system (ZW) by sex-linked markers [37]. There are three screening strategies for sex-linked markers (taking the ZW sex-determination system as an example): (i) based on sex-differences in allele frequencies, (ii) based on sex-differences in heterozygosity, and (iii) based on sex-limited occurences (see Brelsford et al. for specific screening strategies [30]). Generally, in the above-mentioned three methods, XY and ZW have the opposite results [31]. Moreover, we used a custom R script to find the sex-linked loci by association analysis from the Stacks outputs with R-4.1.1 [38].

### 2.5. Confirmation and Assignment of Sex-Specific Markers

The sex-linked loci screened by the above steps usually include false-positive loci. Therefore, we excluded the false positives by BLAST 2.12.0+ and established a local blast database by using the *population.sample.fa* file generated in stacks [39]. Then, we aligned sex-linked loci obtained through the R script to the database using BLAST 2.12.0+. Taking the ZW system as an example, if the sex-linked locus is homozygous with the male sequence alignment result, and the female sequence alignment result is heterozygous, then the sex-linked locus will be retained (the opposite is true for the XY system). In other words, it is preliminarily considered that the sex-linked locus is the confirmed sex-linked locus of *E. multiocellata*.

### 2.6. PCR Validation

To ensure the sex-linked loci from the above step were reliable, we designed PCR primers for sex-linked loci and verified them by PCR amplification. The conditions for PCR amplification we created are an initial 4 min denaturation at 95 °C, followed by 32 cycles of 30 s at 94 °C, 56 °C annealing temperature for 30 s, and 72 °C extension for 40 s, with a final extension of 8 min at 72 °C.

It is noteworthy that there are two ways to perform PCR verification. Taking ZW systems as an example, first, as an approach based on sex-limited occurences, we designed both forward and reverse primers on specific SNP sites of females. To ensure that male bases would not be amplified, the female-specific base was used as the first base of the 3′ end of the primer. To ensure that male bases would not be amplified, we then separated the target sequence by agarose gel (3.5%) electrophoresis. The PCR products were separated by electrophoresis, and we expected that a band could be seen in females rather than in males. Second, in an approach based on sex-differences in allele frequencies and heterozygosity, we designed both forward and reverse primers at the conserved region of sex-linked loci to ensure both male and female individuals could be successfully amplified. DNA sequencing of the PCR products was performed at the Sangon Sequencing Center (Shanghai, China). According to the sequencing results obtained, we expected that at the same SNP sites, females were heterozygous and males were homozygous.

### 2.7. Sex-Linked Markers Assignment

We used the BLAST 2.12.0+ to align the confirmed sex-linked loci with the W chromosome of the female sand lizard (*Lacerta agilis*) (GenBank assembly accession: GCA_009819535.1) and Z chromosome of the Mongolian racerunner (*Eremias argus*) [40,41]. If their e-value was no more than 1 × 10^−25^, the top matches of sex-linked loci were be retained.

After that, two loci that were successfully verified in *E. multiocellata* were aligned to *E. argus*, *L. agilis*, and *Zootoca vivipara* genome assembly (GenBank assembly accession: GCA_011800845.1) by the BLAST 2.12.0+ [39], and then, they were extended by 100 bp before and after their location, a total of 500 bp including the loci were intercepted to construct the species tree. We also used the same method to locate and intercept the contig data of *Lacerta bilineata* (GenBank assembly accession: GCA_900245895.1). Finally, neighbor-joining method was used to construct the gene genealogies for the Z- and W-linked sites by MEGA 7 [42]. The Bayesian inference (BI) analyses evolutionary tree was generated using PhyloSuite v1.2.3 [43] under the GTR + G model. View trees were constructed using FigTree v1.4.4 (http://tree.bio.ed.ac.uk/software/FigTree/ (accessed on 15 May 2023)).

## 3. Results

### 3.1. GBS Data Analyses and Sex-Linked Loci Screening

First, we obtained the raw Illumina reads from the GBS library constructed for 10 females and 10 males of *E. multiocellata*. Then, we de-multiplexed and filtered the raw reads by the process_radtags module in Stacks. Next, after a certain number of low-quality reads were removed, we obtained 656,005,254 reads. Finally, we analyzed the data obtained in the previous step through the de novo_map.pl pipeline in Stacks, and a catalog containing 3,299,907 loci was produced (Supplementary Table S2).

### 3.2. Sex-Linked Marker Screening

We used an approach based on sex differences in sex special to identify 164 ZW sex-linked SNPs (single-nucleotide polymorphisms), and they were located on 53 GBS tags. By comparison, only three sex-linked SNPs with XY patterns were identified and were not located on any GBS tags. In addition, we did not screen any sex-linked SNPs using an approach based on frequency differences and heterozygosity (Supplementary Table S3).

### 3.3. Validation of the Sex-Linked Marker

After eliminating false positives by BLAST 2.12.0+, we retained 38 GBS tags, which were confirmed as sex-linked markers. The 38 sex-linked markers were all female sex-linked markers, and they were screened through the third strategy (Supplementary Table S4).

### 3.4. PCR Verification

**Figure 2:**
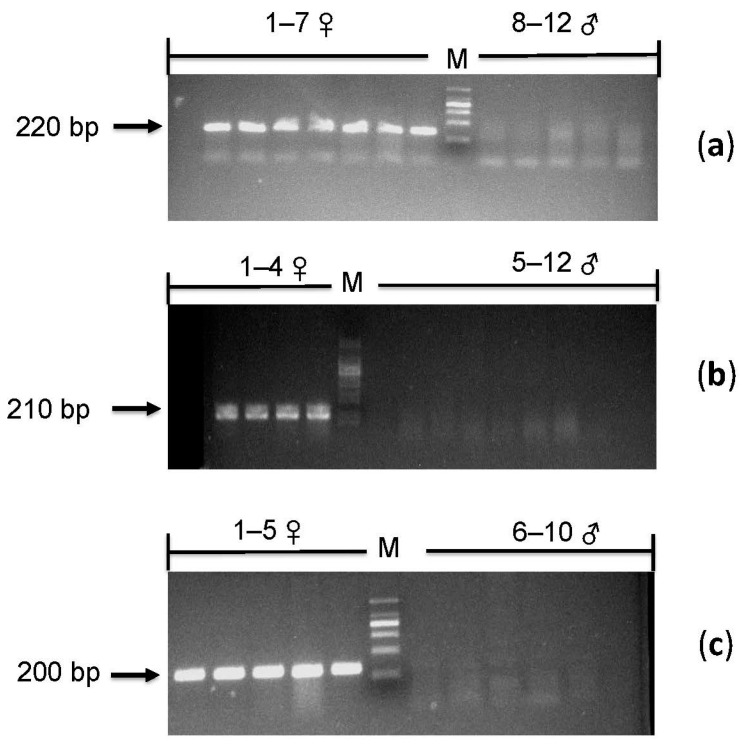
Results of sex-linked loci agarose electrophoresis. In (**a**–**c**), the female has bands, and the male has no bands. This result supports the ZW sex-determination system. For the multiple panels, the samples are listed as follows: (**a**) 1–7♀: Guo822, Guo8735, Guo1792, Guo1696, Guo1160, Guo893, and Guo9003; 8–12♂: Guo9077, Guo5353, Guo8963, Guo8390, and Guo4885; (**b**) 1–4♀: Guo8744, Guo5623, Guo5052, and Guo5072; 5–12♂: Guo4712, Guo5365, Guo5006, Guo5420, Guo8446, Guo8935, Guo8671, and Guo5064; (**c**) 1–5♀: Guo1788, Guo1803, Guo1804, Guo1805, and Guo1807; 6–10♂: Guo1774, Guo1775, Guo1783, Guo1784, and Guo1791. The detailed samples and name of each population are shown in Supplementary Table S1.

We obtained a total of 38 confirmed sex-linked markers, and then, these markers were used for PCR validation, and the first type of primer-design method was used to design the primers; a total of 38 pairs of primers were designed (Table 1). All these sex-linked markers were identified by agarose gel electrophoresis after amplification of 12 samples (including seven females and five males). A total of eight markers were verified successfully. Moreover, all individuals were successfully amplified and showed sex differences after agarose gel electrophoresis; that is, there were bands in females and no bands in males (Figure 2a).

Then, to confirm whether the eight markers verified from the above-mentioned 12 samples of *E. multiocellata* were conservative in other individuals of the species, we selected another 12 samples (including four females and eight males) for agarose gel electrophoresis, and seven of the eight pairs of primers were also verified successfully (Figure 2b).

After that, to further verify the universality of sex-linked markers of *E. multiocellata*, we selected *E. argus* for conservative verification. We used the eight markers obtained in the previous step for cross-validation, and two markers were detected by agarose gel electrophoresis in 10 samples of *E. argus* (including five males and five females). All individuals were successfully amplified and showed gender differences after agarose gel electrophoresis; that is, there were bands in females and no bands in males (Figure 2c). These verified markers can be used as molecular markers for genetic sex identification of *E. multiocellata*.

### 3.5. Sex-Linked Markers Assignment

**Figure 3:**
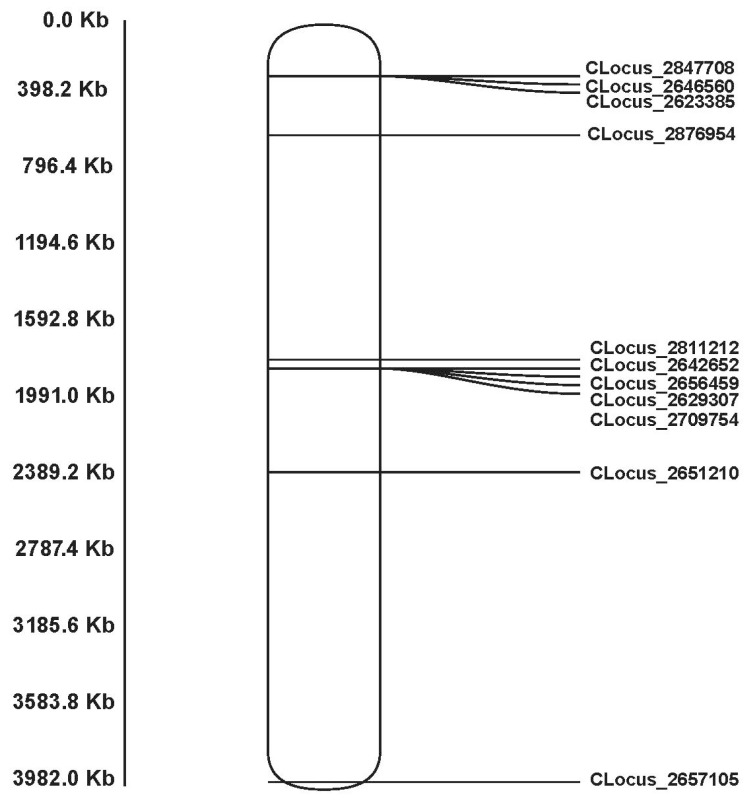
Mapping of the sex-linked markers of *E. multiocellata* to the W chromosome of *Lacerta agilis*. The length of the chromosome based on assembly of *L. agilis* W chromosome. A total of 11 loci were mapped on the W chromosome of *Lacerta agilis*, supporting the female-heterogametic sex-determination system and that the W chromosomes are conserved in Lacertidae.

**Figure 4:**
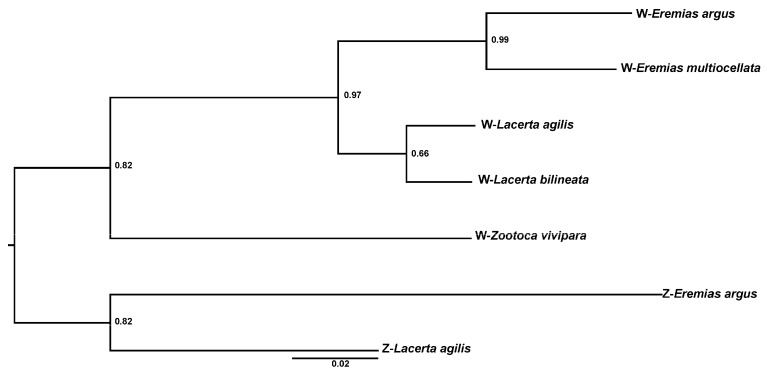
Unrooted phylogenetic tree of one sex-linked marker (CLocus_2847708) based on the Z-linked sequences and W-linked sequences. Bayesian inference and neighbor-joining yielded the same topology. Numbers at the nodes are Bayesian posterior probabilities. As expected, if there was no Z-W recombination, the sequences formed separate Z and W clusters rather than clustering by their species, supporting the conclusion that the W chromosome is homologous in these Lacertidae species.

Finally, 22 of the 38 GBS tags (58%) were successfully mapped to the Z chromosome of *E. argus* (Supplementary Table S5). Furthermore, 20 markers were successfully mapped to the sex chromosome (both Z and W) of *L. agilis*, which includes 11 markers on the W chromosome (Supplementary Table S6). According to the results of sex-linked loci alignment with the W chromosome of *L. agilis*, we made a W chromosome map, which showed that the W chromosome is conserved in in other members of Lacertidae (Figure 3). After that, we constructed a phylogenetic tree of the sex-linked marker for the Z- and W-linked sites based on the obtained sequences of five kinds of lizard. Because of the sequence differentiation between Z-linked and W-linked loci, most loci cannot be successfully blasted. Only the sex-linked marker (CLocus_2847708) can hit on both Z and W chromosomes in *Lacerta agilis* and hit on the male genome of *E. argus*. The W sequences of the sex-linked locus (CLocus_2847708) of *E. multiocellata* and *E. argus* were obtained by female PCR product sequencing. The other species’ sequences of this locus were obtained from homologous sequences on the W chromosomes of each species’ genome by blast. The obtained W-linked sequences for each species were 250–300 bp, and the Z-linked sequences of *L. agilis* and *E. argus* were about 150 bp, respectively. The results indicated that the gene genealogies of Z and W fall into two clusters: one cluster of the Z-linked sequences and another one of the W-linked sequences (Figure 4). That means the sex-linked locus cluster by gametologs and not by species. It implied that the W chromosome has conservation and is homologous in Lacertidae (Figure 4).

## 4. Discussion

In the present study, a total of 38 sex-linked markers were identified by association analysis of GBS data, and the results strongly suggest that *E. multiocellata* has a genetic sex-determination system with ZW. Eight sex-linked markers were successfully verified in the PCR results of 25 samples of *E. multiocellata*, which basically covered the geographical distribution of *E. multiocellata* in China. Unlike other temperature-influenced sex-determination lizards, such sex-linked markers are shared in all studied populations without intraspecific variation [14]. At the same time, two sex-linked markers were successfully verified in 10 samples from *E. argus*. The results indicate that the sex-determination system of *Eremias* is homologous and conserved. To further confirm this point, 38 sex-linked markers were blasted onto the sex chromosome of *L. agilis* by homologous sequence matching, with 20 markers successfully mapped (i.e., 60%). This is consistent with the notion that the sex-determining mechanism and sex chromosomes of lacertid are conserved. 

Regarding the sex-determining mechanism of *E. multiocellata*, researchers have proposed two views: one supporting that it is TSD [8,23] and the other questioning the existence of true TSD in the lizard [17]. Because the former lacks two key experiments, one does not exclude differential mortality between the sexes at a particular temperature, and the other does not consider temperature-mediated sexual inversion. In particular, temperature can interfere with sex ratios but not determine sex ratios. The results are consistent with *E. multiocellata* exhibiting GSD.

The present study showed that the location of 11 sex-linked markers in *E. multiocellata* could be matched to the W chromosome of *L. agilis*. Using several sex-linked genes, 16 species of lizards were found to have ZW sex chromosomes by qPCR [18]. However, such research suggests that the sex-determination mechanism and sex chromosomes are relatively conserved in lacertid lizards based on the sex-linked genes on Z chromosomes. The variation of males and females is different in gene expression but not in the sex chromosomal sequence differences. In this study, the validated sex-linked markers are all based on sex differences in sex-special reads rather than based on sex differences in allele frequency or heterozygosity, which supports the deep sequence variation of Z and W chromosomes. For some studies isolating sex-linked markers by GBS, many markers differences between ZW (or XY) were found to be SNPs, which suggests very nascent sex chromosome variation. Accordingly, the difference between Z and W of the lacertid could be accumulated after a long evolutionary time, so it can be considered that the ZW differentiation began to happen in association with the diversification of the lacertid [44]. In the meantime, both the location of the sex-linked maker and the phylogenetic tree of the Z- and W-linked loci support the conservation of sex chromosomes of all of Lacertidae (Figure 3 and Figure 4). It implied that the Z and W chromosomes were differentiated from their Lacertidae ancestor, and GSD is likely an ancestral state in Lacertidae. For the other species in Lacertidae, if the sex ratio can be influenced by temperatures, it could be temperature-influenced GSD [14]. Even if it is a true TSD, our results indicate that there could be an independent origin of TSD from a GSD ancestor [45]. Nevertheless, there could also have been a turnover event that created a new female-determining factor on a different chromosome or changed to male heterogamety in some lacertids; however, more species need to be studied for confirmation in future research.

Comparative to other squamates, the sex chromosome conservation is constrained in Lacertidae. In other iguanian and gekkotan lizards, the sex-determination systems are relatively diverse [12,14,46]. Some species of Gekkonidae are with both XY and ZW GSD in the same family [47,48], and there are even different sex-determination mechanism in different populations of the same skink species (*N. ocellatus*) [9,14]. Though the sex chromosomes are conserved in Lacertidae, it seems that the sex chromosome did not fall in the deep variant evolutionary strata, as most of the sex chromosome are homomorphic [3,6]. This homomorphy indicates the low differentiation of the sex chromosome and nascent “evolutionary strata” of the sex chromosome [25]. This lower differentiation of the sex chromosome could also explain that how the sex ratio of offspring could easily be influenced by temperature, even with definitively ZW sex-determination systems.

The scope of this conservation seems to be limited in lacertid lizards. As a recent study by Li et al. [40] reported, the Z chromosome of chickens, lizards, and snakes evolved from different ancestral autosomes, and there is more evidence that the male-determining genes *Dmrt1*, *Dmrt2*, and *Dmrt3* of birds are located on chromosome 18 of the Mongolian racerunner or chromosome 17 of the European common wall lizard. Therefore, due to the fact that the chicken and lizard do not share the same Z chromosome region, it can be determined that all reptile sex chromosomes are not conservative [14,40,46].

## 5. Conclusions

We uncovered the ZZ/ZW sex chromosomes of *E. multiocellata* by GBS. The verified sex-linked sequences could be used as genetic markers to identify the sex of *E. multiocellata* and its closely related species. A total of 11 sex-linked markers were successfully mapped to the W chromosome of *L. agilis*. Most species of lacertids have conserved systems, unlike the geckos, which frequently switch, but they do not share the same sex chromosome as other reptiles. There is some indication for future research that Lacertidae has this ancestral character trait (GSD sex determination).

## Figures and Tables

**Table 1:** Primer information of the sex-linked loci of female-specific sequences.

Locus	Primer Sequence (5′–3′)	Annealing Temperature (°C)	Length (bp)
2648560	Forward: CGAAACATTTGACTTCTGACGC	55	298
	Reverse: TCCGACCTTATATGCCTCTTCT		
2847708	Forward: AGCTCTCCCCAATCATCACC	55	298
	Reverse: AGAAAGCAGGAAGGGGAGTG		
2811212	Forward: GTCCAACATCCTTTGCCCAA	55	298
	Reverse: CACCATCAACCTCCTCCCTT		
2811108	Forward: TCTTCTGATGGCCTTGGACC	55	298
	Reverse: GGCATCCAAACATCTCACCA		
2645069	Forward: AGCATCTTCTTCCCTCTCTTCT	55	298
	Reverse: TGGGATCTGGATTGGCTGTT		
2642652	Forward: AGGATGTGGGAGGCTGTTTT	55	298
	Reverse: TGCAACTGTGGACTGAAACG		
2628103	Forward: AGCCTCTTCTTCCACTTCCA	55	298
	Reverse: AGTGTGTGATGGAGATGGGG		
2629307	Forward: TGCCTCCCATCACAGTTCTT	55	298
	Reverse: TACAGCAGGACAAAAGGGCA		

## Data Availability

The data supporting the results of this study can be found in the manuscript. The raw sequence data obtained in this study have been deposited in China National GeneBank DataBase (CNGBdb) and are publicly accessible at CNP0004189.

## Supplementary Materials

The following supporting information can be downloaded at: https://www.mdpi.com/article/10.3390/ani13132180/s1, Table S1: Specimen information and location data; Table S2: The GBS results of *E. multiocellata*; Table S3: The information of sex-linked loci of *E. multiocellata*; Table S4: The identified sex-linked loci information of *E. multiocellata*; Table S5: The sex-linked loci of *E. multiocellata* and 15 (Z chromosome of *Eremias argus*) comparison results; Table S6: The sex-linked loci of *E. multiocellata* and NC _ 046330.1 (W chromosome of *Lacerta agilis*) comparison results.
