# Supplementary material for: Sex-Linked Loci on the W Chromosome in the Multi-Ocellated Racerunner (Eremias multiocellata) Confirm Genetic Sex-Determination Stability in Lacertid Lizards
Source: Animals (Basel). 2023 Jul 3;13(13):2180. doi: 10.3390/ani13132180 (PMC10340011; doi:10.3390/ani13132180)
Supplement: Supplementary file 1 [file animals-13-02180-s001.zip › Table S1.pdf]

Table S1. Specimen information and location data

Sample No.	Species	sex	Geographical origin	Latitude	Longitude
Guo1160	*Eremias multiocellata*	female	Bayan Nur, Inner Mongolia	41.1N	107.87E
Guo1696	*Eremias multiocellata*	female	Yulin, Shanxi	38.39N	19.73E
Guo1792	*Eremias multiocellata*	female	Lanzhou, Gansu	36.1N	103.79E
Guo4714	*Eremias multiocellata*	female	Yulin, Shanxi	38.67N	109.65E
Guo4900	*Eremias multiocellata*	female	Yulin, Shanxi	38.83N	101.01E
Guo5052	*Eremias multiocellata*	female	Gonghe County, Qinghai	36.33N	99.71E
Guo5072	*Eremias multiocellata*	female	Haixi Mongolian and Tibetan Autonomous Prefecture, Qinghai	37.46N	95.63E
Guo5623	*Eremias multiocellata*	female	Dulan County, Qinghai	36.02N	97.75E
Guo822	*Eremias multiocellata*	female	Ordos, Inner Mongolia	39.37N	109.72E
Guo8471	*Eremias multiocellata*	female	Bayan Nur, Inner Mongolia	40.42N	106.99E
Guo8735	*Eremias multiocellata*	female	Wuwei, Gansu	37.31N	103.161E
Guo8744	*Eremias multiocellata*	female	Wuzhong, Ningxia	37.96N	107.1E
Guo893	*Eremias multiocellata*	female	Wuzhong, Ningxia	38.89N	107.71E
Guo9003	*Eremias multiocellata*	female	Wuzhong, Ningxia	38.66N	105.61E
Guo4964	*Eremias multiocellata*	female	Kazak Autonomous County of Aksay, Gansu	39.42N	94.28E
Guo6940	*Eremias multiocellata*	female	Bayingol Mongolian Autonomous Prefecture, Xinjiang	42.44N	86.24E
Guo6965	*Eremias multiocellata*	female	Bayingol Mongolian Autonomous Prefecture, Xinjiang	42.77N	86.32E
Guo2472	*Eremias multiocellata*	male	Lanzhou, Gansu	36.04N	103.94E
Guo2890	*Eremias multiocellata*	male	Wuwei, Gansu	38.62N	103.14E
Guo2945	*Eremias multiocellata*	male	Wuwei, Gansu	37.91N	102.93E

Sample No.	Species	sex	Geographical origin	Latitude	Longitude
Guo4712	*Eremias multiocellata*	male	Yulin, Shanxi	38.67N	109.65E
Guo4885	*Eremias multiocellata*	male	Lanzhou, Gansu	36.49N	103.4E
Guo5006	*Eremias multiocellata*	male	Geermu, Qinghai	37.61N	95.37E
Guo5064	*Eremias multiocellata*	male	Gonghe County, Qinghai	36.27N	100.35E
Guo5353	*Eremias multiocellata*	male	Xining, Qinghai	36.53N	102.02E
Guo5365	*Eremias multiocellata*	male	Dulan County, Qinghai	36.39N	98.09E
Guo5420	*Eremias multiocellata*	male	Dulan County, Qinghai	36.02N	97.94E
Guo665	*Eremias multiocellata*	male	Yuzhong County, Gansu	35.97N	104.17E
Guo8390	*Eremias multiocellata*	male	Yinchuan, Ningxia	38.43N	106.56E
Guo8446	*Eremias multiocellata*	male	Bayan Nur, Inner Mongolia	41.49N	106.92E
Guo847	*Eremias multiocellata*	male	Wushen County, Inner Mongolia	38.46N	108.75E
Guo8671	*Eremias multiocellata*	male	Linze County, Gansu	39.26N	100.1E
Guo8935	*Eremias multiocellata*	male	Zhongwei, Ningxia	37.44N	104.53E
Guo8963	*Eremias multiocellata*	male	Wuzhong, Ningxia	37.52N	107.07E
Guo9077	*Eremias multiocellata*	male	Alxa Left Banner, Inner Mongolia	40.17N	104.72E
Guo1061	Eremias multiocellata	male	Alxa League, Inner Mongolia	40.01N	103.03E
Guo1189	*Eremias multiocellata*	male	Urad Front Banner, Inner Mongolia	41.33N	108.51E
Guo1208	*Eremias multiocellata*	male	Dalad Banner, Inner Mongolia	40.21N	110.01E
Guo1218	*Eremias multiocellata*	male	Xilingol League, Inner Mongolia	42.65N	115.57E
Guo1836	*Eremias multiocellata*	male	Kangping County, Liaoning	42.83N	123.17E
Guo4960	*Eremias multiocellata*	male	Linze County, Gansu	39.26N	100.09E
Guo4965	*Eremias multiocellata*	male	Kazak Autonomous County of Aksay, Gansu	39.42N	94.28E
Guo5092	*Eremias multiocellata*	male	Haixi Mongolian and Tibetan Autonomous Prefecture,Qinghai	37.98N	95.068E

Sample No.	Species	sex	Geographical origin	Latitude	Longitude
Guo6936	*Eremias multiocellata*	male	Bayingol Mongolian Autonomous Prefecture, Xinjiang	42.44N	86.24E
Guo6957	*Eremias multiocellata*	male	Bayingol Mongolian Autonomous Prefecture, Xinjiang	42.77N	86.32E
Guo824	*Eremias multiocellata*	male	Ejin Horo Banner, Inner Mongolia	39.37N	109.72E
Guo860	*Eremias multiocellata*	male	Wushen County, Inner Mongolia	38.18N	107.69E
Guo926	*Eremias multiocellata*	male	Ordos, Inner Mongolia	40.08N	107.73E
Guo1774	*Eremias argus*	male	Chifeng, Inner Mongolia	42.89N	120.34E
Guo1775	*Eremias argus*	male	Chifeng, Inner Mongolia	42.89N	120.34E
Guo1783	*Eremias argus*	male	Chifeng, Inner Mongolia	42.89N	120.34E
Guo1784	*Eremias argus*	male	Chifeng, Inner Mongolia	42.89N	120.34E
Guo1791	*Eremias argus*	male	Chifeng, Inner Mongolia	42.89N	120.34E
Guo1788	*Eremias argus*	female	xilin hot, Inner Mongolia	44.20N	115.92E
Guo1803	*Eremias argus*	female	xilin hot, Inner Mongolia	44.20N	115.92E
Guo1804	*Eremias argus*	female	xilin hot, Inner Mongolia	44.20N	115.92E
Guo1805	*Eremias argus*	female	xilin hot, Inner Mongolia	44.20N	115.92E
Guo1807	*Eremias argus*	female	xilin hot, Inner Mongolia	44.20N	115.92E

Note: we selected 20 samples for analysis (♀: Guo1160, Guo1696, Guo1792, Guo4714, Guo4900, Guo822, Guo9003, Guo8735, Guo822, Guo8471; ♂: Guo2472, Guo2890, Guo4885, Guo665, Guo5353, Guo847, Guo8963, Guo9077, Guo8390, Guo2945). we selected 34 samples for PCR verification (♀: Guo8744, Guo5623, Guo5052, Guo5072, Guo822, Guo8735, Guo1792, Guo1696, Guo1160, Guo893, Guo9003, Guo1788, Guo1803, Guo1804, Guo1805, Guo1807; ♂: Guo4712, Guo5365, Guo5006, Guo5420, Guo8446, Guo8935, Guo8671, Guo5064, Guo9077, Guo5353, Guo8963, Guo8390, Guo4885, Guo1774, Guo1775, Guo1783, Guo1784, Guo1791).
