# Supplementary material for: Sex-Linked Loci on the W Chromosome in the Multi-Ocellated Racerunner (Eremias multiocellata) Confirm Genetic Sex-Determination Stability in Lacertid Lizards
Source: Animals (Basel). 2023 Jul 3;13(13):2180. doi: 10.3390/ani13132180 (PMC10340011; doi:10.3390/ani13132180)
Supplement: Supplementary file 1 [file animals-13-02180-s001.zip › Table S2.pdf]

Table S2 The GBS results of *E. multiocellata*

Sample	Raw Base(bp)	Clean Base(bp)	Error Rate(%)	GC Content(%)	Average depth （X）	Effective Rate(%)	Total-SNP
**Female**							
Guo8735	1,231,840,224	1,231,840,224	0.04	43.77	4.68	100.00	5598458
Guo9003	1,051,442,496	1,051,442,496	0.04	43.01	4.35	100.00	4724808
Guo1696	2,377,806,195	2,377,720,371	0.03	43.73	7.01	100.00	12806942
Guo4714	2,775,717,792	2,775,700,224	0.03	43.19	7.85	100.00	15563024
Guo4900	2,413,617,984	2,413604,448	0.03	42.92	7.71	100.00	13798978
Guo822	1,778,529,312	1,778,524,704	0.03	43.86	6.40	100.00	9372400
Guo893	1,861,386,515	1,861,380,755	0.03	44.05	5.98	100.00	9595964
Guo1160	629,926,848	629,906,112	0.03	42.75	4.34	100.00	3473084
Guo8471	1,993,541,184	1,993,489,920	0.03	44.31	6.65	100.00	10720500
Guo1792	2,173,791,680	2,173,716,800	0.03	43.59	6.96	100.00	11827948
**Male**							
Guo665	2,167,246,944	2,167,224,480	0.04	41.75	7.19	100.00	11898624
Guo5353	1,901,571,264	1,901,489,472	0.03	43.03	6.91	100.00	10419004
Guo2890	2,314,459,294	2,314,437,694	0.03	43.09	7.83	100.00	13147230
Guo2472	2,121,109,799	2,121,087,911	0.03	43.30	7.23	100.00	11831540
Guo2945	1,435,448,448	1,435,259,808	0.04	42.77	5.89	99.99	6541598
Guo847	1,484,464,608	1,484,420,256	0.03	44.28	5.35	100.00	7375550
Guo8963	1,124,268,480	1,124,229,024	0.04	43.63	4.25	100.00	5220292

Sample	Raw Base(bp)	Clean Base(bp)	Error Rate(%)	GC Content(%)	Average depth（X）	Effective Rate(%)	Total-SNP
**Male**							
Guo4885	2,410,947,360	2,410,883,424	0.03	43.13	7.35	100.00	13426584
Guo8390	1,981,593,504	1,981,548,000	0.04	44.56	7.11	100.00	10482638
Guo9077	1,232,690,400	1,232,658,432	0.04	43.42	4.98	100.00	5935992

Note: Raw Base: Initial data; Clean bases: the amount of effective data after filtering; Error rate: the average error rate of bases; GC content: the proportion of bases G and C; Average depth: average Sequencing depth; Effective Rate: Average effective rate of base; Total- SNP: total number of SNPs
