# Supplementary material for: Sex-Linked Loci on the W Chromosome in the Multi-Ocellated Racerunner (Eremias multiocellata) Confirm Genetic Sex-Determination Stability in Lacertid Lizards
Source: Animals (Basel). 2023 Jul 3;13(13):2180. doi: 10.3390/ani13132180 (PMC10340011; doi:10.3390/ani13132180)
Supplement: Supplementary file 1 [file animals-13-02180-s001.zip › Table S3.pdf]

Table S3 The Information of sex-linked loci of *E. multiocellata*

Locus	F count	M count	Criteria for sex-linkag	GBS-tag (T or F)	Seq
482326	7	0	(iii)		TAAAACATCATTCCACTAAAAGTAAACAAAATGGGCAGGGTTCACCAGTCCTTTCACCATAAGCCCTGTGCAATGATGTCTGCTTGAACAACAGGGTTTTCCCCTTTCCTTCCCCTATGCCCCCCCAAAGTTGACAGGGGTGGGG
753445	8	0	(iii)	T	TAAAGACCCTGTAGAAAGCTGTGCTTCTTGTACTCTGACACTATCCCATCTGGATTTTTTTCCCCTTCCATGAAGAAATTTCAAATATATTAGCCTCATCTGCCATGAGTGGCTAATTATCTGATATGCTAATGTGAAAATGATT
1363913	7	0	(iii)		TAAGTCCTCTAGGTAAGTGCTTGTGATCCTAGGCAAGTAAGCCACAAGGACTGGAGATAACTTGTGACATTTATGTAAACCATTCTTGACTTTTTTTTTCTTTCTTTTTTTTTTGAGAAAGACAGTTACAACAAAGTAGCAAATACCCACTTTTC
1821010	8	0	(iii)	T	TAACTTTTTTTTTTTGTTTTGGGGGGGGGGGAGGGAGGCAATCAAATGAAATCCCACAAGCTGTCATTCAGCAAACACTGACAACTACTATATTATGGTATGTATAGGAAGGCTGGGGAACTTATGGCCTTTTGGTTACTGTTGGATTCCAAT
2091823	7	0	(iii)		TAAAGGGAATGCAGTGACGCTACACAGGCAAGAAATGAAACCCCCCCCCTTTTGTGTGTAAGTTGGAATGGTTTGCTTGGAAAGTACTTCAGAGAGTTGTTACCCAGTTTCTGAAAGGGGGCGCAGTGAGTGTACAGTATGCTGAGTCGGAGGGAGAGCTGTG
2280877	8	0	(iii)	T	TAACTCCGAGCCAGTTGGAGTCAGTTGGGAACAACTGTCAAGGGGTGACCGTTAGAAATTTGAATTGGAGGGAGGAGTATAAAAAGGGAGAAGTACCAGTGTGAGAGTTAATTAGAGTTAGAGTGAGAGTTCTGGGAGAGAGTGTGTGGGTGGTAATTG
2496572	7	0	(iii)		TAATGCAAGGACTTCAACTTGCCCGACAGGACTTCCTCTCCCTCTCCCTCTCCCTCTCCCTCTCCCTCTCCTTCTCCCTCTCCCTCTTTCTCTGTAGCCCTGAATCCCAGAAGCCAGTTCTGGCATTTCCCTGATGCTCTGGAGCAGATTGAATGGATGGAGACACAA
2509101	8	0	(iii)	T	TAAAAATTTTTATTATTTTTGAAAAGAACGGTAAGGGGTTTTGCAAAGGTTTGGGCCTGATGGCACTCTTCGAATTGTAAGTAACGTTTCCATGTTTTTTGCGAGACGATCCGTCCAGCGGTGTAGGCGTTCATTTGCCCACAAATTT
2604112	7	0	(iii)		TAAACTTAGGAAGAAAAAAAAAACCACCCTAGTGGCCGAACTATATTCCCTGAAGCAGATGCTCTGCCTTGTCATC

					GGCGCTTCGTTGCTGTCGGTGGCTGCCTGCAAGCGGTTCCTTTGGCGGAAAGGGAAAGGCACTCTGTACATGCTC
2604336	7	0	(iii)		TAATAGCAAAGACAAAAAGTGGTGAGGATAAGGGGGGGGGGGGTGAGAGAGAGATGCTTTTGGGCCAACGAGC<br>AACAGGAAGTTTATATGCAAGAGTCAACCAGCTAAACTCAACTGGAATCTAGCCAGCCCATCTGCCTCCCTCACT
2605241	8	0	(iii)	T	TAAGATCAGAATACAGTGTAAAATTTGACCCTTTGGTCCACATACTGTATGTTGCCAATGTTCCACCTGCATATTATA<br>ACCTTTTTTTCTTCTTTTTTGCCCATTTCATCATACATGGTTTTGCTTCTTCTTGAATTTCAGATT
2605598	7	0	(iii)		TAAACCATCTGGTCCTGGTGATTTTCCTGCTTTTGCCTTTTCTATTGCCCATTGTACTTCATGTGGGGTTATTTTCTCA<br>TTCAACATAAACTTGTTATCCGTTGGGATTTTCTTCAATTCATTCTCTTTTAGATATTTATCAAT
2606222	7	0	(iii)		TAAAAACACTTTGATGACGAGCAGAAAAGCCCCAACAAACGAGCCCTCTATGTGCACATAGCAATATTTTCAAGG<br>CCTCTGTTTGCTGAGAGTTTTCCCAATGGCTGTTTTGTGACTCAGTGAAAGCTATTGCTTTGGTGTCGC
2606425	7	0	(iii)		TAAAAAAATACATTTTAATTTATATTTTTATGATACAACTTTTTTATAATGGAAGATCAATCTCAGAACTGCCGAAAG<br>CCTCCAGTGAAGATGATTCCCAAGGAAGCCAAATAAAACTAAACTTTTGACAGGGTATAAAAATACATGCTACGTG<br>GAGCACATT
2608067	7	0	(iii)		TAAGAAAGTACTAAAGTACACATTCGAGAAAAAGCCAGAGGCCTTCCTACTGGGCATAGTTGGTAAAGAAATAAA<br>TAATAAAGATAGGACATTTTTTAGCTCATTATATATTTTCTCTAGAAGGCCTTCACCACTGGGCAGGTC
2610570	10	0	(iii)	T	TAATTATATTGTAAACCTCATCCCAGAATGGTCTTATTTTTATGCACGTCCACCACATGTGCCATACTGTCCCTACATC<br>CAACTTGCACCTCCAACAGACATTACTACATCCTTTATACATTTTTGCCAATTTGTGTGGTACCA
2612740	7	0	(iii)		TAACAGTTTTTCAAACTCTGTTGCAAAACACTTTTCATCTGTCAGGGACTGACACCATGCAAAACACCACCATCCG<br>ATAAATTTACTCCCTCTGCCATCTTCATTTATAAATAGCCTCTGTATCTTCCATATTGTGCAACATCT
2613373	8	0	(iii)	T	TAAGTACCCACCACCCAAAAGTACAATAGAATTTCCTTTGCATGTGTGGAGCCTTAGGCAATTCTGATGATGAGATT<br>TGGGGCTTCCCTTGGCAGACAGTGAGCTCAGCAATGGTCTCCTCAGGGCATTTCCTTGAGTTAGAGG
2613552	7	0	(iii)		TAAGCACCTCTTACTTGCCTTTTCCCCACACCAACCCACAAGCCCATAGATGTTGTAAGCTTTACTCATAGGAGAG<br>AAGGGACACGGGTGGCACTATGGTCTAAACCACTGAGCCTCTTGGGCTTGCCTATCAGAAGGCTGGCG
2614555	7	0	(iii)		TAATACAAAAGGTCTCAGGAAAGAAGAGGGTGCAATAATTCTAATTCAGCAAGGAATACATCATTATAACCATCGG<br>TTGGGAACCTTTGGCTCCCCGCATATCACTGAACTATAATTCCAATTAGCCCCAGCAAACAAACCAAT
2615254	7	0	(iii)		TAACAAGAAGAACCAGAGGCAGGGGGTCCTCCAAGGACAGGACTGGATGCAGAGGGACACATTTTGAGCTCAG<br>GATCTGAAGGAGAGGACAGGGAGGGGGCAGGTGCTGCAACAGCATCACAGCAGAAGCCTGGTCCCTCTTCC

2615774	7	0	(iii)		TAAAGATAAGCAAACTCCTACACAGAGAGCTCAGTAGCAGAGAACTGGGCAAAGAAAGCTGCATGTTTATTTGAGAAGTGCTGTAGCTCAGTGGTAGAGCATCTGCTTCCCCCAAGTTTGAACCCCAGCAGCTCCAGGTGGAGG
2618458	7	0	(iii)		TAATTCTCTATAAGGCAGTCATATCTGTAAAAATGGGCGCTGCTGTGATCGACCTCACAAAGCTGTTGTGCGGGCAAAACGTTGCCCAAATTTCCAGGGCATTTTGCACTACGGAGTTTTGCGACAACACTGAGAAAGCTCTCT
2619237	8	0	(iii)	T	TAATGTATGCTGCAATAAATCATTTTTGATAAAAGCAAGCTGGCTGCAAAGTGCGGTTCTTGCAGAGACACCCTCTAATTGACTCATAACAACGGATAACGACAGAAACTTATGAGTTTATTTTGGCTCTCTTTCAATGGCTGG
2623261	7	0	(iii)		TAATCTCTGACCTCTCTCTGTTTCTTCCTCCCAAGGCCACTCCTCTACCTTTACCAAAGTTCCTTTACAAACTTTGCTGGCTTCTGGGCCAGTTCTGCCAAACTGACACCAGCAACAATAGAGATAGAATCACAGGATCTGATT
2623385	8	0	(iii)	T	TAAGAAAGCAGGAAGGGGAGTGGCTATACTATTGCTCAAACTGTCCAAGACAGAGCAGTAGTCTGCAGAGGTAGATTGCCTTGGAGCTAGTTAGTGTGGAATGGTGTGCATATCCTTGCACCTTAGGAGCTGATGTAATGGAGT
2624170	7	0	(iii)		TAATCATTCTAAACTGGTTCCATGGGGAGAGCATCTCCCACCAGGTAAACATCTGGATTTTACCCTGCTTCAGGGCTCATATAAAGGACCAAAGTCCTGCCCGCTTTCAGTTAGACCAGTTAGCGAAACTACTAGGAAATGGGG
2624288	7	0	(iii)		TAAACACAGCTCAGACGTGACAAGAATGAACGCCTAACCTCAATGCTCTGAAAAAGCCACCAGTCAACAGCGCCTGTCCAACTCATCAAGTCAACAACACCCATCAACTGCCCCCAATACAACCTCCAACCAGAACACACCCAAC
2624394	8	0	(iii)	T	TAAGAGGTGGGGGGTAAGTTACAGGGGGTGTCCCATATACAGTCATATGGGTGTTCGATTGGGGTAGTTTGGGCCATTTGGCACATGGCCGTTTGGTCCCAGGTCTCAGCTGACATGAAGGGCCTTGTTCAGGGGCAGGCCGTT
2625313	7	0	(iii)		TAAACAGGACTTATGAGTAGAGAGGGCTCAGATTGCACTGCTATCTATCGCTCCATCATAGGAATGAGTATTGCATTGAGAGGAATGAATTTGCAAGTGAGATGTACAGTAATTTATATGTGTGCAAATGTGTTTTGTGCACACTCTCA
2627302	7	0	(iii)		TAAACAGTTCCCAGTCTTTCTCCACTGCTTCTTCACTGAGGTCGCGGATTCTCCCAGTCATCTCCGCGAGTTCCATGTACTCCAGCAACATCATTCTCCACTCTTGTCTTGTCGGTAATTCTTGTGTCTTCCAGTATTTTGCAA
2627643	8	0	(iii)	T	TAAGAGTGATATTTTAATTGGAAAATGTGCATTCTCAAAAGTACAATTGACTCTGACGTCCCAATGTAGCAATATACACAGAGGAGAGTACAGTATTTTCAGTGCCTCATCTGAAAAAGTTGCTTTAGGATATAACAATTACTGCATTAGCCCTTTATGCCAG
2627651	7	0	(iii)		TAAGAACGGTCCTCTGGAACGGATTTTGGTTGTGATTAGAGGTTCCACTGTAATGTTACTAGTTGGTAGCATAGCTATACTTCCTCCCCCCTTTCCACCCCACACATCTTTCTACTTGCCGCCTTCTCCTCCAGCAACCCACTT
2627803	9	0	(iii)	T	TAAGATTACACCTCATAAACTACTTTTGACGACAGGAGGAAAATGTGCCAGGAGTGCATCAGCATGTCACCGACGT

					AGCAGCTCTGCCTTCGTGCAGTCGCAGTGGCTTGTAAATTGCAACTCCCTGCTGTCAAGGGCGCTTGA
2627893	7	0	(iii)		TAAAAAAATAAATAATAATCCTTCCTGGATCACAATACAGTATCATTTACATTTAGCAACATATAGAGAATCCACAAC
					AGAAGCTGTTGGGGAAAACAATTCTGTATCAGGAAGCTTATACTGTTTGATGCTTTATTATGTTTTATATATT
2627947	7	0	(iii)		TAACCCATGTCTGAGGCCCACTGCTGCGCCCCCAGATATGGTCCCGAACGCACGCGCGTAAAAGTGCGCTGTTCG
					TCCGAATGGAGATGGGGAATTTAGCACTCCTGGCTGCGTCACGCTCTTTCCTGCTGCTCACGGTGGACA
2628103	10	0	(iii)	T	TAACTTGCCTGCTTCTTACTTGAAGAGCCTCTTCTTCCACTTCCAACCTGTTCTGTCCCCCACCTTCTTCTCTGACT
					GCTCAGACTCTTCCCCTTCCCTTTGCCCTGGCTCCTGTTTCACAGGTGGCAACAAATAGCCATAAAT
2628230	7	0	(iii)		TAACCTTCTCTTCTCCAGGCTAAACATCCCCAGCTCCCTAAGCCGTTCCTCACAAGGCATTGTTTCCAGGCCTTTGA
					CCATTTTGGTTGCCCTCCTCTGGACACGCTCCAGCTTGTCCGTATCCTTCTTGAACTGTGGGGCCCAA
2628336	10	0	(iii)	T	TAATTTCTTGCGTAGCGGAAGCTTTCAGCTTATTTTGCCAAGAGTGATCAAAAATAAAGGCAGCAACACTGTTTCT
					TCTAATTCTACAGTGTTTATTAGCAAAACTTCAAAACAACAAACAGTATATATATTACAGATAACTTC
2629184	8	0	(iii)	T	TAAGCCCCCCAAAGCGGGAGAATCCCGCAAAAAAATGGGAGGGTTGACAGGCATGCTGGGGAGAGTCCAGAAG
					GCCAGATAGATGTGATGCTCATGGAGCTATACAGAACCCAAATCCTAATAATTAGCAGAAGAGATACATCACATGTC
2629307	10	0	(iii)	T	TAAAAACTTTTGAGCTACTGTGGATGACTGTCCATCACTACCCACAGCTTGTTGTTGCTTCTTAGCTGCCTCCCATC
					ACAGTTCTTCCACATTTCTGTAGTGGCAACCTTGCCCCCATCCTAGTTGTCATAGTCCCTAATGATA
2629531	7	0	(iii)		TAAGAATTCCTTTGGCATTTTAGTCACTGTGTGTTTTTGAATTTACATTATCTACAGTATGTGTTCTTCTTTCCAGCAA
					ATGCCTCTCCAGGACTTGCAGCTTCCCTGGTTGCCTGAAAGAGCAAGTAGGGAGATATTTCATGATGTCT
2629796	7	0	(iii)		TAATAATAGATGATCCTCTCTTCTTCCTCCCCCCCCCTCACTTGCTTTGCATGTGTAACCAAGCTGTGGTCAGATAAT
					GCAGCTGGCCCTTTGTTTGACACATTTGGGGAAGAATGACCCCTGGGACAGGATGCAGCAGTGTGCAAAGCT
2629867	7	0	(iii)		TAAATCCGTATAGACTTACCCATGTGTACAGCAGGGAGGATAAATACCAAGGAAACAAAGCAACTGGTCAGGAAC
					ATGTTTGGGTGTGGAGATGCACTTTGAAGATATGCCTCGTCTTCTGAAGACCTGTAAAGAGGAGCAGGG
2630176	9	0	(iii)	T	TAAGCTTCTGGGCGGGGAACAGACAATCGGTACTTCGAGGTTCTGATTCTGACTAAGCATGTCGTACTTTCTGCTT
					TGCTGTACATAATGCAATAAAGTTAGTTAGTACTACCAGTTGGCTGGTTTATGTCTGCGGACGCTGAT
2631438	7	0	(iii)		TAATTATATTATATTTAGTTTTGATCTCACATCTCTTTATAATATAGAAACTATTACTTATCTGAGGGCCAATGGCTTCT
					ACTCTGGAAGTTTTCTATTGTACATGGGCTTATGTCAGGGGTATCCACTCTTCCCATTATTGTT
2632474	8	0	(iii)	T	TAACAAATAAGGGACTGTGGCAGGAAAGGGGGCCAAAATAAGGAAAATCCCTGGAAATAAGGGATACTTGACAG

					CTATGGCGATGGGGTGAGCTCCCGTTGCTCAGTCCCAGCTCCTGTCAACCTAACAGTTCAAAAGCACTTT
2632491	7	0	(iii)		TAAGTACGGACCTCCAGAACAGATTATGGTTGTAAGTAGACTCACCACTTTAGCTAATGGGGGCTTCCTGCTACCA<br>CCGTGCCGCTAGCACGCGATTTCTGTACTCATCTTGAGGCAAAGGTTGTAACTTGGAGCAACTACTTC
2633125	7	0	(iii)		TAATGTCGGTTCAGAGGTCACTGCTCTCATGGAAATGGAGATGAAATGAAAGACTATGGCAAGGTGGACAGTGCT<br>CCCGCTTGGGTGGCCGTCCAACTTCATGACTCCTTGCACTCCCTCCTTAGGGCTCTTTATTTTTACACT
2634103	10	0	(iii)	T	TAATAGAGATATATAATAATCAGAAGAAACATACAAGTGGAGTCATGATGCCCAGTCTGAGGATTGTGGTCCTGAA<br>CACAATGGCCAGGCTCAACAGCATGTATGAATTCTGATCATAAATCCCACATGATTCCTACAATGCAG
2634483	7	0	(iii)		TAATCCAGTAGTTTCTGCTTTCAAAATAAAGTGCTCTTCAGCTGTAAAAATGACCACCTCCAATGAGAGGATCAAA<br>GATAATGCGTCTGATTTCTAGAGAGGGCTTTTCTTAGCCTTGGAGCCCTTTCATGGACTGTCGAATGA
2634869	7	0	(iii)		TAACAGGGATGCGGGGGGCCCTGTAGTCTAAACCACTGAGCTTCTTGGGCTTGCCGATTGGAAGGTCAGCGGTTT<br>GAATCTGGGTAAGCTCCTGTTTCTGTCCCAGCTTCTGCCAACCTAGCAGCTCAAAAGCACACCCGTACA
2634927	7	0	(iii)		TAATAATAGGCCTGTTTCTCATTATTTCCCCCATACACACACACACACACACACACACACACGCACACACACACGC<br>ACACAGAGAGAGACACAAGTTCTGGTGGACTCGGGTGGACTTGTGGGTATCTTGGCGACGTTTCGACGAGGTCTC<br>ACTCCTCATCTTCAGGCTG
2635844	10	0	(iii)	T	TAACAAATAATTCGCCAACAGGCTAAAATCTGACCCACAAACAGCACATAGGTGGCACCAGAGCGTACCAATACA<br>CCCACCCCTCCCACAACACACCAATGATGTTGCTAGTAATCTTAGCAACATTGCTCACAAACCTCATGC
2638060	8	0	(iii)	T	TAATTTGTGATTTGTGATTGATGGAGTCGGCATGGAGAGAAGAAAGAAAAGACTCTCAGATTCCCCACTTTCCCAA<br>ATGTTGCAGTTCTTTTATCAGGTAGCTTATGTCCAACAAATCATAGTTACCTCACCAAAGACTCTTAT
2638191	7	0	(iii)		TAAAACAGGAGGAGGAGGAGTTTGGACTTGATATCCCACTTTATCACAACCCTAAGGAGTCTCAAAGCGGCTAAC<br>AATCTCCTCTCCCTTCCTCCCCTACAACAAACACTCTGTGGGGTGAATGAGGCTGAGAGACTTCAGAGA
2641161	9	0	(iii)	T	TAAAAAAATATATAACCACCTTTTAGAATGGGAAACTAAAGACGAGTATGTAAAAGAGGCAATGATCAGGTGGGCC<br>CAGGATATGGGCAAAAATATTATGTTAGAAGAGTGGCAGACTTTATGGAATAAAGGACTGAAGTTTACA
2641543	7	0	(iii)		TAATTTGTTGTTTATTGACTTTATTTACTTCCTTCAAATTTTAGGGGTGGCATTCCATCATTCTCCTGGCAATCATGGA<br>TCATCATCATCATCATCATTATAAGTTTGTATACATCAATATAGGGTCGCCAGGAAGAAATAATGATG
2642383	7	0	(iii)		TAAATCCTTCAAGACTAAAAAGCCATTGATCAGTTTTTATAGATATTTAGTATCAGTAAATCAATTTGAACTACAAAA<br>TCTAAGAATCCAAAATGTAATACAAATTCCTCTAATCCAAATCCTATTGCTGAGCAGACCACCTTCA

2642522	7	0	(iii)		TAACAGGAACCGCACACACAGCTGGAGATCAAGAAGTAGGACTTGTCTCCTATGGGCAACTGTTGATTTGGGTTT<br>ACAAACTGGCCCTAACTTGGAGACGCCACCAGCCCCTGCCGACGAACAAATGACTATGGTGCTAACTGG
2642652	8	0	(iii)	T	TAAGTCACACAGAGCCCTCTGTAACGCCTCTGGGGTCCCATGCTCCTTTTATTTTATTTTATTTTTGAAAGGAGGAT<br>GTGGGAGGCTGTTTTTAGTTTGGTGGTTCATTCTGTCTCTTTCCATCCCTCCCTGTAGCGGGGTTATGAGCT
2644282	8	0	(iii)	T	TAAGGGATATCGTGTAGGGCAAGAAAGCAATGGCAGCTACAGTAGTTTTATTAGAGGTAAATGTGTAATGGACTGT<br>AGCTGATAGAGGACTTTTTATTTTTGTGATGAGAAGCAAATGGTGAGCCACTGTGTGAGACAGAATGG
2645069	10	0	(iii)	T	TAAAGCATCTTCTTCCCTCTCTTCTTTATTTATAATTGCCTATTTGAACATACAAAACTGCCTTATATTTAGTCAGAAG<br>ATTGATCTATCTAGATCTGTGCTGACTGGCAGCAGCTTTTCTGGGCTTCAGGCAGCAGACATTCC
2645688	7	0	(iii)		TAAATTGTACCACTAAACCATTATCTTGAAGATGATAAACTGTTTCATTCCACAACAGGTCCATAGACACCGCACAA<br>TTTCTGACAGGAAATTAGTTCCTCAGTCAGACAGAATTTCAAAAGGAAATCCTAGTCTACAAAACAC
2647001	8	0	(iii)	T	TAAAAAAATCCTTTGTATTTTTCTGTCTTCCATCTAACCAAACTTTCTAACAGTAGAGCCTCTGAAGGTAGGAGCCC<br>TGGCTTCAATCTGTGGGTAACTCTGTGGGATCTGGATTGGCTGTTGCATTTTGCATGCAGAAGGTCC
2647600	9	0	(iii)	T	TAAAAACCTGGTATTTGGGAAAATTACTTTCTTGATGCATAGGAAACTGAGTCAGTTTTACAGAAACCTGGTAAAG<br>TACAGGAAATGTCTGGGACAGGTAGGGCTCTGTCGAGTGTGTGATGGAGATGGGGGGGAGGAGAAGCT
2647780	7	0	(iii)		TAACGTCAACCATCGGTTTAGGCTTTTCTTTCTGGTACCGAACAGTTGCAAGGCATGAGCCTAGATTTTGCTTAGG<br>AAAACAAGACAAAACAGAAAATGGTGCTATTTACTTCCAAATGAAAGGCTTTTGCATTACCACATTAC
2648122	7	0	(iii)		TAAAAACTTTGATAAACTGTAGCTTTTCAGACATTATTGGCTAGTGAAATAATGGGAGAGGACCAGGATGATGGTT<br>GATTCCTTCCATGACTTTCATTATAATGCAAAACGTTCTAATGTTCCATATCATTATCATATTTCATT
2648560	9	0	(iii)	T	TAACTTCTGGGTTGTCGGCATACAAAACCCGAAACATTTGACTTCTGACGCATTCACCAAATGAGGTTCCACTGTA<br>TTCCAAATGGAGGGAAATAAACAATGCCAGCTACATGGTTAGCATAGTTTCCCTCCAATTCTGGGGAC
2648724	7	0	(iii)		TAACAGATAAAGTTTCCTTTTACAGCACATATATAGTCTCTTCACAGAGATTTCCACATTCTGTTGTAGAGAGACTTA<br>TACAACCATGATCCTTTCTTCAGTTAGCTAATTGGCTGACTACAGATCCTGCCATTCCCTTTTGGA
2650231	7	0	(iii)		TAAAAAGAAAACGGCATAGTTGAGCCTCAGATAATCTCCACTCCGCTGCCTGAGCCAGCTCTGGGAACCTGTGAT<br>CCCTTATTTTGGCTGCTGTGATCCCTTATTTTCCAAGCCAAAACTTGACAGCTATGCTTTTGCCCACTC
2650492	7	0	(iii)		TAACACAGGGACTGGGCCGCAAAGTGATGACAACCACATGTCATCCTGAGCCACAAGAGCGGGCTTCCCCCAGTA<br>TGCATCTATGGTTTGCTGGGTCTTGGTTTAGCCAGCATTTCTAAAACTAATTATGGAACTTGAGTATGC

2650643	8	0	(iii)	T	TAAGTTGAGAATGACTTAAGTTGAGGTACCACTGTATCTTCTTTTGAGGTACAGTATATGATTTAGGCCTTGCCCAG CGGTGTCTTATGTCTACTTATAAATTACTATCCATGTTGCAGGGAAAAAACCTCTTGAATTATATCCCTGTAACATGG AGACTAACACA
2651210	8	0	(iii)	T	TAAAAGTAGCCCCATGTAGATTACATTGCAGTAGTCCAAGCGGGAGATAACCAGGGCATGATAGGGTCTCAGCCTG CATACAAGATGGAGCTGGTAGACAGCTGCCCTGGACACAGAATTGACCTGACAGCTGTGAGTCCAAAA
2651709	10	0	(iii)	T	TAACCAAAATAAAAATAAGGGATATTGTGTAGGGCAAGAAAGCAATGGCAGCTCTAGTAGGTTTATTTGAGGTAAA TGTGTAATGGACTGTAGCTGATAGAGGACTTTTTATTTTTAGTGATGATGGTGATGATAACTGCGCAG
2652133	10	0	(iii)	T	TAAACTGAGCTGTGTCATTCAGGGTGCACACAGGATTCATGGAGTTCTCATACACAACAAGGTCCTTCAGATCCTT CTTCTTGGAGGAATCTCGGGCAGCCTCACCAACTTTCTGAACATGGACTGCTTTGGTGACCCCAGCAT
2652284	8	0	(iii)		TAATACCTGCCCAGGGACTGCCCAGGGACTGCCCAGGGACTGCCCAGGGATGGAGCTTCGAGCTCTTGTTTTTCC ACTTCTCATATTCTCTTTGTTCCTCCATCATGGCCCTGAACACCTTTGAGATGGTCCAAGGGGAGGAGGGAGATGG GTGAATATCCCACAT
2652720	8	0	(iii)	T	TAATTTCCTGTATCCTACACTATCTCCTTTGCATTCTGCTTGCCTTCTCTCCCCCGCGATTTGTAAGGCCTCACTGGA CAGCCACTTTACTTTCTTGCTTTCTACCGACGTCGCGTCTGACTTGTCCCTAACAATATCCATGGC
2655031	7	0	(iii)		TAAACTGAAATGGTAAATCCATGGGAATCGTAGGAATGCGTGGAATGAGGACATCTTCACCTTTGTTGTGTTGGAG ACTGATTTGGGGTTTCTGGTTGAGGTCTTTCCACCACTGAGAGCCTTTTGCATTGAATCTGTTGAGAA
2656459	8	0	(iii)	T	TAACACTTTGAAATGGCAGGTGCCACCTCTATATGCTTTGACAATGAGCTATGGGCCCCTTTCCTGCAACTGTGGAC TGAAACGGGGGAGGGGGAACCCTTGCGCCCAAATGAGGGATATAAAAACAAGGAAATTTATCTGAGG
2657105	9	0	(iii)	T	TAAAGTATTTGGGTTTGATAGGGGATCTCATAGCTCTATGATAGTTGCTCATCAGAAACTAAAGAACACTATAAAAT GCTGTTCTGCCAAATACAGAGGTGGTTCTCAGAGGTATCAGGACAAATAAACCAAGCAGACAGAAGG
2657292	10	0	(iii)	T	TAACCTGAAGCACCAGTTTAGCTAATGGGGCCTCCTGCTGCCGCTGTACCGCTATATTGCTGGCACGCTGTACTGCT GGCACACGGTTTCTGTTCTCAACTTGAGGCAAAGAACTCACCTTGAGGTAACTACTCCCATACATAC
2657524	8	0	(iii)	T	TAAGGCATCGCAAAAAGTACAAGCGCTGCTGGGCCTTCTTCACTAGCGACTCAGTGCTCCACAACCAGACCAACC TGTTTGTTAGTTGTACTCCCAGGATTTTATGCTAGCAAACATTTCCATTGGCAGGTTGTCAATAAATAG
2658173	7	0	(iii)		TAACTAAAAGTAGAAATTAGGAAAAGTAGAAAAGTAGAAATTCTGGAAAGATTTAGAGAGTGCTTTCTGAGCACT GGGGTGTGTGTGTGTGTGTGTGTGTGTGTGTGTGTGTGTCAAAAGAAAATACAGTTGTACCTCTAGTTGTACCTTA

					GCTTCTTCAGGTTACAT
2660240	7	0	(iii)		TAATGGAAGAAATACCTGTAAGCCTCTGTAGATGCTGGATGTAGATGTTTATGTAGATGTGGAAAACTGGGCAAACGTAGCAGCTTCTGTTCATTTTCCCCTTGTTTTTTTCATATACCATTATGATACTTGCTGTGTGGCACACCTGGA
2660482	7	0	(iii)		TAAAGGATTTTGGGGGGGGGGAGGAGTTGGGAGAAATTAGCTGGAGAGGGGGAGAAAGGGGTTCCAGATATGGAGGGGGGGGGTTCCTTTTACTCTCCCTGGATCTACAAAGGGGCAGCTGGGAGGGGAAGGGAGGTTTCTTGGGGGAGGG
2665400	7	0	(iii)		TAACCCGAAGTACCACTGTATTGGCAAACCCTGGAAAAGCAACAACATCCTACAGTTGCATCACAAGGTAACAAAGTTGTAAAGAGCTGAGTTTCCTGCTCTGTTGTTCTTGCTCTGACTTACACCTTTCTAGGACAAAAGAAAGTT
2666913	8	0	(iii)	T	TAATTCTCTCAAATCCATTTCTGGACCTGCCATCGATCTGCGGCCAGAATTGAGGATGCACGCATGCAAACATGACAATTGTGTTCTGCAGCTTCACATCTGCATCGTTCTCAACTGTTTCAACACAACAGGCTCTCGGTTGTG
2668377	7	0	(iii)		TAAGTCCTCCTTGTTCAAGGTATTCTGATGTGACCCGAAAAATTTCTTCTCCTGTTGTTTTTTCTGGCAATGCCTTGCAAAAAAAAGAAGTTTTCTCTAATTGCATCACCATCCACAAAACGCACATTGGCCAAGAGTTGAGCATGTCCACTGATATCA
2669513	7	0	(iii)		TAAGCAATCATATATGTCCGACAGTCAACTGATTGCGGCAAGTAGAGGGAGGAAGCATGTTCAAGAAACCAAAAGAATGACTACATAGAAAAAGGAAAAACCTGTCTTTAGAGAATACTTTAGCTTATTGATGCGGCACGTGCT
2675858	7	0	(iii)		TAACCACATCAAAATTACATAAATTTATTTGAGCAGAAACAAATGCTTGGATAAACAAAAATGTTTTCAGTTGCTTGCGAAAGCATCATCCTCAGGATATTTTCTGTGTCTCTTGGGGCAGACTGCTCCAAATTGCAGACTATACCATTGAG
2676964	7	0	(iii)		TAAAGCGTGCGTACACTGCAGGTAGCTGAGGGATCGTTGTAGCGCTGAGGTGGGGTAACTGTACTGACTGCTTGCGCTCGTGGCTTCCTTGCAAACAATGTTCTGTGCAGAACTTATAGTCAAATCTGATAGATTTATCTGCCC
2686458	8	0	(iii)	T	TAATACCCTCCCTTCACCCAGAGGGAAGGTTACATTGTGTAAAAACACAAGAAGAAACACAATAAAGTTACAGTAGACTGTAATAAAAGCATCAGCAAATCAGTATGAAATAATGGAGGGAGTAGATCATTTCATATCAACCCT
2691342	7	0	(iii)		TAAGCCATTTCTATAAATTGTGATACCGCCATTTTTTTGTCTAAGGATTTCATGGCATCCAAAATGGGAAGACCACTGTTTTTGGAAGGACTTGCCATCCTACATTCTTCTAGAATTGACCTCTTATGAATTTTGTTGGGTTTGA
2692930	8	0	(iii)	T	TAAACCCTCCATTTGGGAATCCCTTGCAGATAACTGAAGTAGCTGAAGACAGTCAGGTCTTTTCTCCACACCAGTGACCAGAGGAGGAATGACCCCTGGGGGGAACACCAGAATGAAGAAATGCCATTATGAACCTGCAGGAGC
2693823	9	0	(iii)	T	TAACAAAATGACCATTGAATACAAATATGTCCTGATGCCAAACCGCTTGTGGCACTGCAGTGTCTATCTACAGGAC

					CACTTTTTAGCTGAAGGGTATGGCACAAAGAAAACCAGCAAGCATGCTGCAGCAGACGAGGCACTGAA
2695650	8	0	(iii)	T	TAAGCGACTTTGCTAGCATAGATTCAGCTGGGACCCTGAGTTCCAGTGATGAACCTGTGAGATTCAAAGCTGCAA
					ATTCTCTGGAAATCAAGCTGGAGAACTCCCAGATTGCAAATGTGATGCATGGGAGGAGGGAATGAAATC
2695803	7	0	(iii)		TAATGTTCACATAAAATGCAGGTGGTGCTGTGGTCTAAACAACTGAGCCTCTTGGGTTTGCTGATCAGAAGGTTGG
					CGGTTCGAATCCCTGCGATGGGGCAAGGTCCCGTTGCACAGTCCCAGCTCCTGTCAACCTAACAGTTC
2697440	7	0	(iii)		TAACTTGGAGAGGCGTGGTCTGCGTCTGAGCGTCTGACGATGTTCCTGCCTACGCTCCCCTGCTGACGGGACCTG
					CACCTCCCCCTCTCCTGAACTGGAAGTGGACATTGGCTGTGAAATCGGTTCTACTGCTCTGGGATGCTG
2700196	7	0	(iii)		TAAGGGAGATCTCGACAGTAGATCGGACTGAAATTCAAAGAACAGCAACTTGGAAAGCTATCCTCACTCTCCCAG
					GCTTCCATCAGATGATCCCAGGCCAGCCACCAAGCTCTCTGTGTGGTCTCATGCAAGTCACTCTCCCTC
2701758	7	0	(iii)		TAACCTCCTTTTACTACTATTCATGAAAATAAAAGCAGTAGCATCTACTATATGGAATCTACTTTGGTCCCCAGCTCT
					TGATCTGAAGAAGTGTGTATGGGATCAGTTTGCAGAATAGTAGAGAGATAAAATGGAGGAAAGAGA
2703351	7	0	(iii)		TAATTCTTATGACATATTGTTGAAGGTTTTTACCGTGTCCATGAACATCAGGATTGCCTATTTATCTGGTCTCATTTCT
					AAGTACTCCAGTATGTCAATTACCATTCTTGTGCTACTGTGCATTTGTATCCCTGGCAAGAAGCC
2703674	7	0	(iii)		TAACCTGAGGCACCACTTTAGCTAATGGGGCCTCCTGCTTCCGCCACGCCGCTGGCGCACGATTTCTGTACTCATC
					TTGGGGCAAAGTTTGCAACTAGGAGCAACTACTTCCAGGTTAGCAGAGTTTGTAACCAGAAGTGTCTG
2706132	8	0	(iii)	T	TAAGTCTCCTTCACCAGTATTGCCTTGTTGAAGCAGTTACCACAATATTATTTCGATACCAGTATTCAATGATATGGC
					AGTTTATTACACAACCCTACTAGCTGGGAACTCCTTATTGTGTTCAGTACAGTCATACCTTAGTTG
2709754	9	0	(iii)	T	TAAAAGATTACAGCAGGACAAAAGGGCAGGGGAGGTCTCACAATAATGCTGGACCAGCCTAAATTCAACAGCGA
					CATGCCAATATGGACTTTGCCAGGACTGTATGCCCTTGAATGAGCAACAGGGTCACCTGCCACCAATCCC
2710219	7	0	(iii)		TAAAATGCCCAACTCCAAATTCACAGCCTTCTGGGTTCTCAGCCAGATGAATGACCAGCAGGTGCCCAGAAGCAG
					TGAATATATTTGGAAAGGACAGAACTAAAGTCACCACTGCGTCCCAGTCTGTGAATCCAGGAAACATACA
2710492	7	0	(iii)		TAAGTCCATATATCATTGAAAGTGAAAAGACTATAAGAGGAAGGTCCGACCTTATATGCCTCTTCTTTGAGGCTGCA
					TGAAGATTTGCAGTGTTAGAGCAGAGATTTTGGAGATGGTAAGAAGGAGGCATTCCAATAATGTGCT
2711760	7	0	(iii)		TAAGGCTACGCAGTAGTTATTCCTGTGCAACTCTTCTGGACAGCAACATCCTAACGCTTAGGGTAAAGAAAGACTG
					CATCCACAGGTTATGGGCCCAGATTCTGAGGCTTCCAAGTTGTGGTGTCCAGAGTTGGTGAGGTCGTG
2717683	7	0	(iii)		TAAGGCTGTCCCGCATTGCAGTAGTCCAAGCGGGAGATGACCAGAGCTTGCACCACTCTGGTGAGACAGTACACA

					GGCAAATAGGATCTCATCCAGTGTACCAAATGAAGCTGGGAGAGAGCCACCCACCTTCAATGCAGACAG
2718013	7	0	(iii)		TAATAGGATGTTCCTTATGCGGTTCTGCTTCTGCCTAGCAGTTAGGTCAGCACTGTCTTTTTCTGTTGTTACTGAAAGAAGAAAAAGAGGATCCCACAGTGTGGGAAAGATTATCAAGACCAAAGAGGAGGTGGATCTTCCCAC
2718166	7	0	(iii)		TAAGTGCTTTACTAATAACACTGTGCATTACAGAATGAGTCTGACAACCACATTTCCAGCTGTGTTTCCTTGCCGTAAACCCATGCACCACCTCACCAGTAGCAAGCACCCCTCTCTTTTCACCCACAGCCCTGCCTGTGGTGGTTTTTTCATACCCC
2719220	7	0	(iii)		TAAATACCACTTTCTTGGCTCATTACATACGCTTTTATATCTTTTATATTTTTTCGAACAACTGCACACACCACCCAGGTGTTATCTTGTTTACCAGACAGGGTTGCTCTTTAGACTGTATCAAGTTCAGTCAAAGGGGAAAGA
2722314	7	0	(iii)		TAAAAGTCTCAAAACTTCAGCTAGCTTTTCTCCAAAGCTTTAGCTCTCCAAAACAGCCACTTTGCCTCCCAGGACCCAATCCTTACTCACTCTTAGGATGTGTCCGAGGCTCTCTCCGTTTTCTTCACTGGCGCCCTTCCACTGTCTGATT
2748810	7	0	(iii)		TAAGCAATGGAGACATTTCCTAATTGGAGCCAAGCATAGAGTGCAGGTGTGCACGGATCACAAGAATCTGGAGTACTGGAGGACGGCTAAGATACTGAACAAGAGGCAGATACGATGGGCAGAGTTCTTTGCGGACTTCAATTT
2749663	7	0	(iii)		TAATAAAAATCGAACTCCAAAACTTAGGCTGAAGTGAACGCGAAAGCCTCTGACTAAGTTCCTTCAGAAAAATCATGAAAACGTTCCTTACCATTCAAAGAGTGCCAACGTGCCCAAAGCTTTCAAAACCTCCTTACCGTTTTTTATAATATATAA
2751846	8	0	(iii)	T	TAAAGACTGCTATATCTGAAGAAGCAGACTCCTCTACAAATGCTTAGACCACAATAAACATGTTAGTCTTTAGGGCAGGGGAAGCCAGCCTGATGCCTTCCAGAGGCTGTTGGACTACAACGCCCATTTGTCTCAGCTGGCATG
2752760	7	0	(iii)		TAAATTGAAATGGTAAATCCGTGGGAATCGTAGGAATGCGTGGAATGAGGACATCTTCACCTTTCTTGTGTTGGAGACTGATTTGGTGTTTCTGGTTGAGTTCTTTCCGCCACTGAGAGCCTTTTGCATTGAATCTGTTGAGAA
2753130	7	0	(iii)		TAAGTTGGCATGTTTTCTCTAACCATAGTAAAGAATAGCCACAGTTCTGATGATCCAGCAAACTGTGAATAATATAAAACACAAACAAATTTCTGAATTCCAGCTTGTAACTACTGTATACCTGCAAAAAGGAGGGAGCAATCCAAGAATTG
2753926	7	0	(iii)		TAAGAATCTGATGCTCTGCTGACTGTGCATATCCCAGTTATCTTGGGAGAAAATGGTTAGTGTTAGTTACAAAAAGTTTCCCCCATCACCACAACCTTGCTTTATGGAGGCTCAGTATTTTACCCTGTGAGAACTTTGTCTCTG
2758178	7	0	(iii)		TAAAGGGGTCAGCAATCTGCTTGTGCCACCTCCATGTTCCCCCTTCCCGCCCTGCGCTCCCCTTCCCAGCCGCGTTCAGCTGCATTGATGTGGGGCATGGCGGTGAGAAACACCATCAACCTCCTCCCTTCCCCCCATCCTTCT
2758804	7	0	(iii)		TAAGATGATGTGATTTATAGCTCTTTGAGAGCTAGAAAAAAATTGCATTGAGACAAAGCCACAGGCTGAGTTTGCG

					ATCCTATTGGGAGCATGTCTTTGCTTATTTCCAAGCAAACATATTTGGATTGTGCTGCAAATACATGC
2761861	7	0	(iii)		TAATGACTGCTGTAATTACAAGTCATCTTTTCTCTTTGGGTGGTCGCTGCTGGGACTTGAACCACTGTCCCCCTCCACATCTGCAAAGAAGGGATCTACTCTGTGAGTAAGGGTGAAGAAGCTTGTTTTGAGTTCTTCTAGTTAGAG
2764875	7	0	(iii)		TAACTAGGCAAGAAAAATGGATTTCTGGACCAAAGCTCACAATCTGAAAGAGATTTATTAGAGAAAAATGAAAAATTATTGTCTAAAATGTATAAGCTAACATAAGAACATCCCTGCTGAAACAGGCCACAGCCCATCCAGTCCAG
2766984	7	0	(iii)		TAACATTGAGTCCGTAAGTGATTGTGGAGGCTAATTCTGGATCCAAACATCCTTCCACAGTGGGGACATAGGTTTCTGGGTGAGAATTGATCATGAGGAGGGTTTGCCAAGCATGCCTTCCTCTTATCTCATTTCTCCCTTTCG
2767727	7	0	(iii)		TAAGCAGCTTGTATGCAAATACTGTAGGAACAAGATAAAACCAAGACAAGCCTCAAGGGATGACTAAAGGTGCTGCTCTTTCAAGTAATAGATTTTGCCCAGAATTGAGAGATACACTTTAGGAATGCCCAAGAGTAATTGTAA
2770340	7	0	(iii)		TAATGACGTAAGTTGAGGTACCACTGTATCTTGAATACCCATGGGTAATGGTGAAGAGAAACCAAGGCAAAGATGCACAACCATACAGCTCACACAGATGCCTTCAGATACAAGGAAGTGGATGTTCTTTGTTCTTTTGGCCCT
2775401	7	0	(iii)		TAAGGGCCAGACTTGAGTGAGCTCAGCTGGCCATAGCCCCACATCTGTATGGAGTTTGGATTTACACCAAAATTGCAGCAGCATAATTTACTCTGGTTGGCTTCCTGTAGTTTGGATTGTTCAGATGTTGCTGGACTACAGTAC
2781689	7	0	(iii)		TAAAGTCTGCAAACTGCGTACCATGTGAATTTACTTGGTTTTGTCATTTTTCCCTCCATGTCAATGGCCAAGCAGGGCAATTGTGATACCATAACTCACCATGTGATCAAGATGGTGTAAGTCGTAAGCAGCTTGCAAATGTGT
2785097	9	0	(iii)	T	TAAGTCATTGTCCTCACAAAATGGAAGCATGGTGAGGACTAAATGCAAATCAAACTCAAGAAGAACGGGCATAGGCGAATGAACAAAGGAGACAAAGGATGGCTCAAATGCATGCCAAAGAAGCAGCAGAGCAACGCACAGCCA
2794446	8	0	(iii)	T	TAAAATGCTAAAAAGCAAAGGTCTATACAATGTTTGGGGCGAAAAAAACCCAGGTGACACTAGCCACACATTCCAGTCTGGACGTCATGGCACAATGTCCAGACTAGACAGCATTCTTCTCACTCAGGGTCTGATGCCCTTGGTGGAATCAA
2803012	7	0	(iii)		TAATGGGAAAGACTTACTAGATGAGGGAAGGAAAGTGGAGGGGAGGGAGTTTACTAGGGATCATATGTAGATATTTGAAGATATATACAAGTTTTGGTGAATTCAGGTCTTTTCCCGTGTAAGTTTGGGGGTATCTTGGTGACG
2807588	7	0	(iii)		TAATGTTCTCGAAGGTCTTCTTTCCCTGCTTTTTCCTCGTATCTTCTTTTCTTCACAGTAGTTGTTAGTAAAGGTGTCTTGGAGTACAATAGTGTGTGTCATGTCTCTTTTTATGAGTTGTTCATTTTACAGTTCAGTTTTGTT
2809142	8	0	(iii)	T	TAAGCGTCTTTGCGGTGCTGGATTTGAGCATGAGGTTCATAAGCACTGTTGCTAGTGTTATTAGCAACAGCATTGTTGTGTTTGGGTAGGGTGGGTGTCTTGGTACGCTCTGGTGCCACCAATGTGCTATTTGTATGTCAGGAT

2811108	8	0	(iii)	T	TAACTTAGTGTGAATGGAAGCAATTCTTCTGATGGCCTTGGACCATCATTTGATGAACTACCATCAACTGTTGGAGCTGCTCTCATCAGAAGGATCACTGTACATATGTTCATTAGTCCTAGATGTCTCATAACGTGTGATGCT
2811212	8	0	(iii)	T	TAAACTTTTTATGTCCAACATCCTTTGCCCAATCTATCATAACAGATTTTACTTGTTCATCTTTCATATGCCACTCCAGCAATAAATTATACATTTTGGAAATATTCTTAGTCTTGGAACGCTTGGCAACTCTCACCTCGCTGGGCT
2812086	7	0	(iii)		TAACTGGAGGATGAAACAGAGAAACCACCTCACTGGACTTTTCTTGGCTTACTGCCACCCATAGCTACTCTCTCTGGGAAGGAAGTGAGCTATGATTTTGCAATCGTATCTGTGCAGTAGGGAGGCAGAAGAGTGGAGGAAGAA
2818973	7	0	(iii)		TAAGCCTCTAGGCAACCTGAGCTACCGTGTAGATACTGATATCACATGAGGACGTCATGCCTCTTATTGCAAGAATGGGGAACCTGTGGCCTTCCAACTGCCACTGAACTGCAACTGCCAGCACTCTTGACCATTGGAAATCAAGCTCCAACAA
2835896	7	0	(iii)		TAAGGCTGTCTTTTTGTGGTTTTGGGATAGAGACTATTTTGGAGTGGGTCCAGGTTTTGGGGATGGGTCCTGTAAGGGAGTCAGGGGAAGAGAAGGCAGAGGGCAAAAGCGGGAAAGAGGGAGAGAGGGAAGTGCCAGGGCTAT
2836578	7	0	(iii)		TAACACAGCAAGCACCACCAGCATTCCCCAACCCCACACCTCCACATAGATCCCCATGCAACACCAGTCTGGACGACACTAAATCACACTACCCATCAACCCACAATTCACCCCTACCACTGACCACTTCAGAACCAAGCAAAC
2843274	7	0	(iii)		TAAGGGGTGTGGGGAGAGTTACAGGGGGTGTCCTATATGTGGTCATATAGGTGTTCATTTGGGGTAATTTGGGCCATTTGGTGCATGTCCGTTCGGCCCTAGGTCTCAGCTGACATGGAAGGCCTTGTTCGGGGACAGGCCGTT
2847708	8	0	(iii)	T	TAAACAGCATGGGGGATAAAATCAAACCCTGAGGAACCCCACATTGAAGGCTTCACAGGGCTTCACAGCTCTCCCCAATCATCACCCTCTGGAAATGACCATCCAAGTAGGACTGGAACAACCACAAAGCTGGGCTCCCTATCC
2869181	7	0	(iii)		TAACTCGTCCTGGTAAATTTATACCTATGCTACTCCTGCCCCACAGCGCTTAGCTTTGGAAGACAGATCAACCTGACTGCACAAATCATTGCATGGTCACACCATCAAATCTGCTACTTGGGGTTTGGAGAGAGCAAAATCCAA
2871145	7	0	(iii)		TAATGGAGGAGAGAGCTATTGGCTTCTGAATCCCAGTTGCTGCAAACCTCAGGAGGGGGGGGAGTTGTGTTTTGGGGTTTCCCATTGGGGCATCTGGTTGTCCCTTGTAAGAACAGATGCTGGCCTAGATGGGCTTCTCTTGGGT
2872615	7	0	(iii)		TAACAACTCCTCCAGTTCATAGAAGAGCTGGCAATCCAGTTGCCAACCCTTTTCCTGGTAGTAAGAGATGACACAATTTGGTCAATTCCTCCTTCCCACACCTGAGCCACCTCCTACATATGTCCAGAGGGCCTTCCAACAATC
2873202	7	0	(iii)		TAAATCTCCCAATAATATAATTTTATAGTCCAAATAATCAGCCAAAGTCTTATCCAACTGCTTATAGAATTCAGCTTTTCCATCATTTGGTGCATAGATACCAGCCATCAAAATCTTTTCGCCTTGTTGGACCGTTTCCACCAG
2874027	7	0	(iii)		TAAAAAACAAACACATGAGGCGTGCGTCCCCAGACCCCTATCTACTTCCAGAATTGTAAACAATAAAATGAACTGT

					TCAAGACACTGAAATGCAGAATATAAGCACAGTAACTTTCACAGTACGTATGGCCAGCTACTGCTGTA
2875107	7	0	(iii)		TAAATTATATTTAGAGTCCTTTTCCTTTTGAGCCATGTTTTGATCTTTTTCTCTCTCTGAAGCAGCCAAGGCCAGTTCCAAACCACAAATATTTTCTTACATAAACATCCAGTGTTCCACTATTTCAAGGTCACTCAGAGATAC
2876301	8	0	(iii)	T	TAAGCCCTGTATACCTGATGGAGTGTGTCTTCCCCCATCATTCAGCCTGGACACTGAGGTCCAGCGCTGTCAGGGGTTCACCCCAGAGTAGCAGAGTGATCAGTGAGGCTGAGCAGGAGGACAAGATAGAGGAAGAGGAGTGGG
2876954	8	0	(iii)		TAAGAAATGGGAGTGCCTGACCACCTCATCTGTCTTCTGAGAAATCTCTATGCAGGACAAGAAGCTTCAGTTAGAACTGGATATGGAACCACTGACTTCTTCAAAATTGGGAAGGGAGTATGACAAGGCTATATATTGTCTCCCTTTTTTTTTTTTT
2877631	7	0	(iii)		TAAACATATGGAACTGAAAAAGAGAGAATTAGACTTCTTAGAAATCAACAAAACAATGATAAACATTCCATACCTAAAACAGCACTTCTACAAATATTTGGGGGGGGGGGACGGCACGACTCCAGGCTATTAGCAAATAGGTGTAGGAAA
2883610	7	0	(iii)		TAAGATCAAGTTCCCTATAAAAGGCAGGCATTTGAGGGAGGGAGCCTCATCACAGACCCAGGGCTAGGGTGCTGTGAGTGGGTTTGTGTGTCCTCGCTACCCATTTGAGGGGCTCCTGGGAGTAGATTGGGGACATGCTGATTT
2899017	7	0	(iii)		TAAGCAGAGGTTGGATAGCCATCTCTCATGGATGCTTTCGTTGAGATTCCTGCCTTGTAGGAGGGTTGGACGCGATGACTCTTTGGGTTCTGCCCAGCGCTACAGTTCTGTGATTCTTCCCTGCTTCTGATTCTAGCCTAATCT
2946768	7	0	(iii)		TAATCTGAACTCTTCGGTGCCGTACCGCACCCAGAGCTCAGAAATTGCTCACTGTAATGAAATTCCTTGTCGCAAAATGCGTCTAGAACAATGGGAGTGTCAAACACTGATGGAAACTACCTGCGTTTAGTATGTGCCTTACTG
2977889	7	0	(iii)		TAAAATATAAAAGGCACTTTTTTTTACTTTTAGATAACACCATGGCAAAAAAAAAAACCAGTAGCTCATTATTGAATTGAATTGAATTGATTATTTGTACCCTGCCCATCTGGCTAACAGTCTGGTCCTAGTGGAAGCAAATGTTATTCT
2981102	7	0	(iii)		TAAATATGCAGATGATATAACTGGTGGGTCAGATTCAGGAAGAAAATGGGGGGCTGTACTGAGAGGAAGGAAGAGTCTAGTGAATTGCTGCAGGAGAAATAATCTTGGGCTGAATGTGGAAATACCAGAGAGATCGTTGTCGAT
3000569	7	0	(iii)		TAAACTCCCATGATCCCTAGGTAACAGGACCAGCAGTGAGGGATGATGGGGAGTTGTAGGTCAAAATATCTGGAGGGCCAAAGTTTGCCTATGCCTGCCTTTGAATGACTGCAAAATAGCTGCAAGAAGACTTTGGTGGCAGCA
3017866	7	0	(iii)		TAAGTGTTTGCTGGCAGGGAAAAGGAAAAACACTTGGTGACCCAACCTGCCCAGCGAAACAATTTTTTTCCGCCACACCCTCCTGGCCTGAAAACACAGCCTTCCACTATCCTCTGACCCATAGGACAAATCACCTATATGCCC
3028454	7	0	(iii)		TAAATTCACTGCAAGCAACTGCCTTAGGGAGGAACTAAACATGGACAATGTGGGACTGCTTTTGAAAACCACCACTTCAATACCATGTCATATTTTCCCCCATTCTTGCCAGTGGCAGCTGATCGTCATGTTGAGCAGAAGTGGA

3057829	7	0	(iii)	TAAAGCACATACACCAAAGAACCATGTGAACTCTATTTTGTTATGGGTGCTGGGAGTGGGAACAATACATGCAATA<br>TATTGGAGTGGTAAACTACAGTTCCCAGGATTCGGAAGGAACAGTGTGCGTAGGCAGCTCCGCATTTC
3122315	7	0	(iii)	TAAACTACAAGGCCTGCTCATTACTCAATACGAAATACTGTATACTGTATATTGCTCAAGGGTACGCAATAATTGTGC<br>CTAACATAAAAGGCAGTTGTAAACTTGTGACTATTGTAAATTGTAAATACAGTGGAAGCTCAACAA
3167280	7	0	(iii)	TAAGTTGAGCTTTACGGCCTCATGTTGAGCAAGGTGCCCAGGGCTGTTGGATGAATTGGCTGCTAGTTGAAGCAG<br>CCTCAAGTTGAATAGAATAGAATAGAATAAAATTTATTATTTGTACCCCGCCCATCTGGCTGGGTTTCC
3213775	7	0	(iii)	TAATTTGTAAAACCAAATAATACAGGTTCTACTACAGTCAAAGGTAGAACACAGGAAAGGCAAGCTATGTGGAGC<br>AAAACCAACAAAACTATGGGCTAATCATCATGATGGCAGAATTGCACCCTACACTTATAGGTTGAACAT
3306623	7	0	(iii)	TAAATGGCAATTCTCAGAATGCTTGAGGCAGCTAACAAAGCAATCACAAGTAAGCCTTCAAAATCCCAATACTAAA<br>AACATCTTCACAATAAAAATAAAAATAAAAAAAGCTGAGCAGTTGCTTCACCTGGAAGAGCTGACTTGAATACA
3326811	7	0	(iii)	TAATCATCTGAGGGTCAATGGCGCTGTGGTCTAAACCACTGAGCCTCTTCGACTTGCCAATCAGAAGGTCGGCGGT<br>TTGAATCCCTGTGACGGGGTGAGCTCCCGTTGCTCGGTCCCAGCTCCTGCCCACCTAGCAGTTCAAAA
3506757	7	0	(iii)	TAAGTGACGTTCGGTCAGCCGTAGAGGAGGGCTGTGTTTCTGTTGTTTGAAGTAGAAAGCAAAGCCAGTCTCCTC<br>AAAGTCTGGGGAGTAATCCTTGTGTAAATTGCCTCTACTCTGTAATGCAAACTGAAATCCAGGACCACC

Note: Col, the nucleotide site within the catalog locus, reported using a zero-based offset (first nucleotide is enumerated as 0); F count / M count, we split samples into male and female: males were assigned to M count, females were assigned to F count; Criteria for sex-linkage, the approaches screened for sex-linked loci confirmed, validation of sex-linked markers, 'F' and 'M' indicate true and false, respectively.
