# Supplementary material for: Sex-Linked Loci on the W Chromosome in the Multi-Ocellated Racerunner (Eremias multiocellata) Confirm Genetic Sex-Determination Stability in Lacertid Lizards
Source: Animals (Basel). 2023 Jul 3;13(13):2180. doi: 10.3390/ani13132180 (PMC10340011; doi:10.3390/ani13132180)
Supplement: Supplementary file 1 [file animals-13-02180-s001.zip › Table S4.pdf]

Table S4 The identified sex-linked loci information of *E. multiocellata*

Locus	Seq
1821010	TAACTTTTTTTTTTTTGGGGGGGGGGGGAGGGAGGCAATCAAATGAAATCCCACAAGCTGTCATTCAGCAAACACTGACAACTACTATATTA<br>TGGTATGTATAGGAAGGCTGGGGAACTTATGGCCTTTTGGTTACTGTTGGATTCCAATNNNNNNNNNNGTCAGGGATGATGGGAGTTGAGG<br>GGTTGCAAGAAGGCAGCAATTCCCATTCCCACCTGCATTCATTGCAATCAGTGCTGTTTCCATTCTGCTGCAGTTTGGGTTCTGCCAAACAC<br>TATGTTATTTGTCTCATTGTCTGTGAATTA
2280877	TAACTCCGAGCCAGTTGGAGTCAGTTGGGAACAACTGTCAAGGGGTGACCGTTAGAAATTTGAATTGGAGGGAGGAGTATAAAAAGGGAG<br>AAGTACCAGTGTGAAAGAGTTAGAGTTCTGGGAGAGAGTGTGTGGGTGGTAATTGNNNNNNNNNNTTTTGGTCTTGGGTGAGGCTGGCTA<br>GGTTGGGAGATAGGAGAGTGATAGGCTTTACTGTTTTAGTTCCTACTTTATCTTAGTCCCTTTATGATCCAACCCTATTCATTTCCATTCCCCTA<br>TCTGTTATCTTTTTATTATTCTTA
2605241	TAAGATCAGAATACAGTGTAAAATTTGACCCTTTGGTCCACATACTGTATGTTGCCAATGTTCCACCTGCATATTATAACCTTTTTTTCTTCTTT<br>TTTGCCCATTTCATCATACATGGTTTTGCTTCTTCTTGAATTTCAGATTNNNNNNNNNNATTTCAACAAATGCCGAGACACTGGAACCTCCAG<br>TAATGAACTCAATCCGTTCCAGGGGGGCCTACCCACCTAGAAGCAGGCGTATTTCAGCCTAGTACCAAAACATTGCTTGGTCTCCGTTTTCC<br>TCCCTAACAAAAATGTAAAAAAGCTAAGAAATACAAATATGTACATATATTGATGCAGAAGATTTTA
2610570	TAATTATATTGTAAACCTCATCCCAGAATGGTCTTATTTTTATGCACGTCCACCACATGTGCCATACTGTCCCTACATCCAACTTGCACCTCCA<br>ACAGACATTACTACATCCTTTATACATTTTTGCCAATTTGTGTGGTACCANNNNNNNNNNCCATTTTATATACATTTTCTTTTAGATCATAGTTG<br>GGTGTAAACTTCAGTCCTTTATTCCATAAAGTCTGCCACTCTTCTAACATAATATTTTTGCCCATATCCTGGGCCCACCTGATCATTGCCTCTTT<br>TACATACTCGTCTTTAGTTTCCCATTCTAAAAGGTGGTTATATATTTTTTTA
2619237	TAATGTATGCTGCAATAAATCATTTTTGATAAAAGCAAGCTGGCTGCAAAGTGCGGTTCTTGCAGAGACACCCTCTAATTGACTCATAACAAC<br>GGATAACGACAGAAACTTATGAGTTTATTTTGGCTCTCTTTCAATGGCTGGNNNNNNNNNNATAAGAGTCTTTGGTGAGGTAACTATGATTT<br>GTTGGACATAAGCTACCTGATAAAAGAACTGCAACATTTGGGAAAGTGGGGAATCTGAGAGTCTTTTCTTTCTTCTCTCCATGCCGACTCCA<br>TCAATCACAAATCACAAATTA
2623385	TAAGAAAGCAGGAAGGGGAGTGGCTATACTATTGCTCAAACTGTCCAAGACAGAGCAGTAGTCTGCAGAGGTAGATTGCCTTGGAGCTAGT<br>TAGTGTGGAATGGTGTGCATATCCTTGCACCTTAGGAGCTGATGTAATGGAGTNNNNNNNNNNGGATAGGGAGCCCAGCTTTGTGGTTGTTC<br>CAGTCCTACTTGGATGGTCATTTCCAGAGGGTGATGATTGGGGAGAGCTGTGAAGCCCTGTGAAGCCTTCAATGTGGGGTTCCTCAGGGTTT<br>GATTTTATCCCCCATGCTGTTTA

2627643	TAATTGGAAAATGTGCATTCTCAAAAGTACAATTGACTCTGACGTCCCAATGTAGCAATATACACAGAGGAGAGTATTTTCAGTGCCTCATCTGAAAAAGTTGCTTTAGGATATAACAATTACTGCATTAGCCCTTTATGCCAGAATACTGCAAGTCATATTTATTTGATCCCCAAACATTGCGCTTCTTATTCTTGATTGTCCTATGCCTGTGAAATATATTGTTAGCACTTGTTGAACGTTTGGTCCTATTTTTATTTACTGTCTAGAGAAGCTGATCAGGCTGTTTTGCTAGCTGTTA
2628103	TAACTTGCCTGCTTCTTACTTGAAGAGCCTCTTCTTCCACTTCCAACCTGTTCTGTCCCCCACCTTCTTCTCTGACTGCTCAGACTCTTCCCCTTCCCTTTGCCCTGGCTCCTGTTTCACAGGTGGCAACAAATAGCCATAAATNNNNNNNNNNAGCTTCTCCTCCCCCCCATCTCCATCACACACTCGACAGAGCCCTACCTGTCCCAGACATTTCCTGTACTTTACCAGGTTTCTGTAAAACTGACTCAGTTTCCTATGCATCAAGAAAGTAATTTTCCCAAATACCAGGTTTTTA
2629184	TAAGCCCCCCAAAGCGGGAGAATCCCGCAAAAAATGGGAGGGTTGACAGGCATGCTGGGGAGAGTCCAGAAGGCCAGATAGATGTGATGCTCATGGAGCTATACAGAACCCAAATCCTAATAATTAGCAGAAGAGATACATCACNNNNNNNNNNCACATGTCTTGTAAGTTGAGGAGATGCATGTTTATGAAGGGGGGAGAGAGAAAGTCTGCCCCCAATCAGCCCACTTTTGCCTCGTCCTGCCCACTGCCAGTATGTGTGTCTCCCAATAACCTTCCCCACAGAAATGAACCCTTAACCCCCAAAATGTTCCCCACCCTTGGCTTA
2629307	TAAAAACTTTTGAGCTACTGTGGATGACTGTCCATCACTACCCACAGCTTGTTGTTGCTTCTTAGCTGCCTCCCATCACAGTTCTTCCACATTTCTGTAGTGGCAACCTTGCCCCCATCCTAGTTGTCATAGTCCCTAATGATANNNNNNNNNNGGATTGGTGGCAGGTGACCCTGTTGCTCATTCAAGGGCATACAGTCCTGGCAAAGTCCATATTGGCATGTCGCTGTTGAATTTAGGCTGGTCCAGCATTATTGTGAGACCTCCCCTGCCCTTTTGTCCTGCTGTAATCTTTTA
2630176	TAAGCTTCTGGGCGGGGAACAGACAATCGGTACTTCGAGGTTCTGATTCTGACTAAGCATGTCGTACTTTCTGCTTTGCTGTACATAATGCAATAAAGTTAGTTAGTACTACCAGTTGGCTGGTTTATGTCTGCGGACGCTGATNNNNNNNNNNCACAATCACCGCACCAGATATTCTCTTTTGCCCTCCTAGACTGTACTCTAAAACATTTTTCCATTTCTTGTTCAATCTCTGCTCCTTCTATTTCCAACCATTGAGCCAGTTCTTCAACCACTCTTTTCCTTTTTTTCTTTATTA
2632474	TAACAAATAAGGGACTGTGGCAGGAAAGGGGGCCAAAATAAGGGAAATCCCTGGAAATAAGGGATACTTGACAGCTATGGCGATGGGGTGAGCTCCCGTTGCTCAGTCCCAGCTCCTGTCAACCTAACAGTTCAAAAGCACTTTNNNNNNNNNNAATAGGTTCCGCTCCAGCAGGAAGGTAACAGCGTTTCCGTGCACTCTGGTGTCGGTGTTCTGTCATGCCAGAAGCAGTAGTCATGTTGGCCACATGACATGGAAAAACTGTTTGAGGACAAATGCTGGTTTGCGACTGGACTTAACTGTCAGGGGTCCTTTACCTTTTA
2634103	TAATAGAGATATATAATAATCAGAAGAAACATACAAGTGGAGTCATGATGCCCAGTCTGAGGATTGTGGTCCTGAACACAATGGCCAGGCTCAACAGCATGTATGAATTCTGATCATAAATCCCACATGATTCCTACAATGCAGNNNNNNNNNNGCCATGGATATTGTTAGGGACAAGTCAGAC

	GCGACGTCGGTAGAAAGCAAGAAAGTAAAGTGGCTGTCCAGTGAGGCCTTACAAATCGCGGGGGAGAGAAGGCAAGCAGAATGCAAAGGAGATAGTGTAGGATACAGGAAATTA
2638060	TAATTTGTGATTTGTGATTGATGGAGTCGGCATGGAGAGAAGAAAGAAAAGACTCTCAGATTCCCCACTTTCCCAAATGTTGCAGTTCTTTTATCAGGTAGCTTATGTCCAACAAATCATAGTTACCTCACCAAAGACTCTTATNNNNNNNNNNNNNNCCAGCCATTGAAAGAGAGCCAAAATAAACTCATAAGTTTCTGTCGTTATCCGTTGTTATGAGTCAATTAGAGGGTGTCTCTGCAAGAACCGCACTTTGCAGCCAGCTTGCTTTTATCAAAAATGATTTATTGCAGCATACATTA
2642652	TAAGTCACACAGAGCCCTCTGTAACGCCTCTGGGGTCCCATGCTCCTTTTATTTTATTTTATTTTTGAAAGGAGGATGTGGGAGGCTGTTTTTAGTTTGGTGGTTCATTCTGTCTCTTTCCATCCCTCCCTGTAGCGGGGTTATGAGCTNNNNNNNNNNCCTCAGATAAATTTCCTTGTTTTTATATCCCTCATTTGGGCGCAAGGGTTCCCCCTCCCCCGTTTCAGTCCACAGTTGCAGGAAAGGGGCCCATAGCTCATTGTCAAAGCATATAGAGGTGGCACCTGCCATTTCAAAGTGTTA
2644282	TAAGGGATATCGTGTAGGGCAAGAAAGCAATGGCAGCTACAGTAGTTTTATTAGAGGTAAATGTGTAATGGACTGTAGCTGATAGAGGACTTTTTATTTTTGTGATGAGAAGCAAATGGTGAGCCACTGTGTGAGACAGAATGGNNNNNNNNNNCTGCACAGCAATGCCACTATTTTCAAGTGTAGTAATTTGGTATGAGATCCAAAGTGGCACGGAATGTGTAAATTTAGAGAAGAAAAATGTATGAGACTGTGGTAAAAGGAAAGTCAGAGAAATGGTTTAGTTGGGATTTTTTTA
2645069	TAAAGCATCTTCTTCCCTCTCTTCTTTATTTATAATTGCCTATTTGAACATACAAAACTGCCTTATATTTAGTCAGAAGATTGATCTATCTAGATCTGTGCTGACTGGCAGCAGCTTTTCTGGGCTTCAGGCAGCAGACATTCCNNNNNNNNNNGGACCTTCTGCATGCAAAATGCAACAGCCAATCCAGATCCCACAGAGTTACCCACAGATTGAAGCCAGGGCTCCTACCTTCAGAGGCTCTACTGTTAGAAAGTTTGGTTAGATGGAAGACAGAAAAATACAAAGGATTTTTTTA
2647001	TAAAAAAATCCTTTGTATTTTTCTGTCTTCCATCTAACCAAACTTTCTAACAGTAGAGCCTCTGAAGGTAGGAGCCCTGGCTTCAATCTGTGGGTAACTCTGTGGGATCTGGATTGGCTGTTGCATTTTGCATGCAGAAGGTCCNNNNNNNNNNGGAATGTCTGCTGCCTGAAGCCCAGAAAAGCTGCTGCCAGTCAGCACAGATCTAGATAGATCAATCTTCTGACTAAATATAAGGCAGTTTTGTATGTTCAAATAGGCAATTATAAATAAAGAAGAGAGGGAAGAAGATGCTTTA
2647600	TAAAAACCTGGTATTTGGGAAAATTACTTTCTTGATGCATAGGAAACTGAGTCAGTTTTACAGAAACCTGGTAAAGTACAGGAAATGTCTGGGACAGGTAGGGCTCTGTCGAGTGTGTGATGGAGATGGGGGGGAGGAGAAGCTNNNNNNNNNNATTTATGGCTATTTGTTGCCACCTGTGAAACAGGAGCCAGGGCAAAGGGAAGGGGAAGAGTCTGAGCAGTCAGAGAAGAAGGTGGGGGACAGAACAGGTTGGAAGTGGAAGAAGAGGCTCTTCAAGTAAGAAGCAGGCAAGTTA

2648560	TAACTTCTGGGTTGTCGGCATACAAAACCCGAAACATTTGACTTCTGACGCATTCACCAAATGAGGTTCCACTGTATTCCAAATGGAGGGAAATAAACAATGCCAGCTACATGGTTAGCATAGTTTCCCTCCAATTCTGGGGACNNNNNNNNNNAGCACATTATTGGAATGCCTCCTTCTTACCATCTCCAAAATCTCTGCTCTAACACTGCAAATCTTCATGCAGCCTCAAAGAAGAGGCATATAAGGTCGGACCTTCCTCTTATAGTCTTTTCACTTTCAATGATATATGGACTTA
2651210	TAAAAGTAGCCCCATGTAGATTACATTGCAGTAGTCCAAGCGGGAGATAACCAGGGCATGATAGGGTCTCAGCCTGCATACAAGATGGAGCTGGTAGACAGCTGCCCTGGACACAGAATTGACCTGACAGCTGTGAGTCCAAAANNNNNNNNNNGGGAGTCCTCCACACCTGCCCACCCCCTTGAACAGTACCTCTGTCTTGCCAGGATTCAACCTCAATCTGTTATCTGCCATCCATCCTCCAACCTCCTCCAGACACGCACACAGGACCTTCACTGCCCTCACTGGTTCTGATTTA
2651709	TAACCAAAATAAAAATAAGGGATATTGTGTAGGGCAAGAAAGCAATGGCAGCTCTAGTAGGTTTATTTGAGGTAAATGTGTAATGGACTGTAGCTGATAGAGGACTTTTTATTTTTAGTGATGATGGTGATGATAACTGCACAGNNNNNNNNNNTGGCATGGAATGTTTAAATTTAGAGAAGAAAAATGTATGAGACTGTGATAGAAGGAAAGTCAGAGAAATGGTTTGGTTGGGATTTTTTTACTGTAATTGCCTTAGCGAGGTGTGGGCAGGGGGGGGGGCTGGATCGATACCTTTA
2652133	TAAACTGAGCTGTGTCATTCAGGGTGCACACAGGATTCATGGAGTTCTCATACACAACAAGGTCCTTCAGATCCTTCTTCTTGGAGGAATCTCGGGCAGCCTCACCAACTTTCTGAACATGGACTGCTTTGGTGACCCCAGCATNNNNNNNNNNTTCAGTGCCTCGTCTGCTGCAGCATGCTTGCTGGTTTTCTTTGTGCCATACCCTTCAGCTAAAAAGTGGTCCTGTAGATAGACACTGCAGTGCCACAAGCGGTTTGGCATCAGGACATATTTGTATTCAATGGTCATTTTGTTA
2652720	TAATTTCCTGTATCCTACACTATCTCCTTTGCATTCTGCTTGCCTTCTCTCCCCCGCGATTTGTAAGGCCTCACTGGACAGCCACTTTACTTTCTTGCTTTCTACCGACGTCGCGTCTGACTTGTCCCTAACAATATCCATGGCNNNNNNNNNNCTGCATTGTAGGAATCATGTGGGATTTATGATCAGAATTCATACATGCTGTTGAGCCTGGCCATTGTGTTCAGGACCACAATCCTCAGACTGGGCATCATGACTCCACTTGTATGTTTCTTCTGATTATTATATATCTCTATTA
2656459	TAACACTTTGAAATGGCAGGTGCCACCTCTATATGCTTTGACAATGAGCTATGGGCCCCTTTCCTGCAACTGTGGACTGAAACGGGGGAGGGGGAACCCTTGCGCCCAAATGAGGGATATAAAAACAAGGAAATTTATCTGAGGNNNNNNNNNNAGCTCATAACCCCGCTACAGGGAGGGATGGAAAGAGACAGAATGAACCACCAAACTAAAAACAGCCTCCCACATCCTCCTTTCAAAAATAAAATAAAATAAAAGGAGCATGGGACCCCAGAGGCGTTACAGAGGGCTCTGTGTGACTTA
2657105	TAAAGTATTTGGGTTTGATAGGGGATCTCATAGCTCTATGATAGTTGCTCATCAGAAACTAAAGAACACTATAAAATGCTGTTCTGCCAAATACAGAGGTGGTTCTCAGAGGTATCAGGACAAATAAACCAAGCAGACAGAAGGNNNNNNNNNNAAATCAGCATGTCCCCAATCTACTCCCAG

	GAGCCCCTCAAATGGGTAGCGAGGACACACAAACCCACTCACAGCACCCTAGCCCTGGGTCTGTGATGAGGCTCCCTCCCTCAAATGCCTG<br>CCTTTTATAGGGAACTTGATCTTA
2657292	TAACCTGAAGCACCAGTTTAGCTAATGGGGCCTCCTGCTGCCGCTGTACCGCTATACTGCTGGCACGCTGTACTGCTGGCACACGGTTTCTG<br>TTCTCAACTTGAGGCAAAGAACTCACCTTGAGGTAACTACTCCCATACATACNNNNNNNNNNATGAAGACAAAGGAGACAGGGACTTGAA<br>CCAGCAAGCTCTGCCATTGGGGGAGGAAGTGCTGAGTCCTGTCTGCGCTGACAGTCCTCCCTCCGCTCTCCATGCAGGCACTTTTATTGATC<br>ACCAGATCACCAGAACACTCATTA
2666913	TAATTCTCTCAAATCCATTTCTGGACCTGCCATCGATCTGCGGCCAGAATTGAGGATGCACGCATGCAAACATGACAATTGTGTTCTGCAGCT<br>TCACATCTGCATCGTTCTCAACTGTTTCAACACAACAGGCTCTCGGTTGTGCATTCCACTATGACCCTGCTGTTGATTATAGCTCAAGCCATC<br>ATGTTCTCATCGGCACTATGACTGAAGTGTGTCATTACTGCAGAGCTCTAAAATTTCCTAAAGAAACTCAAGGAATGTGTTGTGCCGCTGGA<br>AAAGTTA
2657524	TAAGGCATCGCAAAAAGTACAAGCGCTGCTGGGCCTTCTTCACTAGCGACTCAGTGCTCCACAACCAGACCAACCTGTTTGTTAGTTGTAC<br>TCCCAGGATTTTATGCTAGCAAACATTTCCATTGGCAGGTTGTCAATAAATAGNNNNNNNNNNATCGACAACGATCTCTCTGGTATTTCCACA<br>TTCAGCCCAAGATTATTTCTCCTGCAGCAATTCACTAGACTCTTCCTTCCTCTCAGTACAGCCCCCCATTTTCTTCCTGAATCTGACCCACCA<br>GTTATATCATCTGCATATTTA
2692930	TAAACCCTCCATTTGGGAATCCCTTGCAGATAACTGAAGTAGCTGAAGACAGTCAGGTCTTTTCTCCACACCAGTGACCAGAGGAGGAATG<br>ACCCCTGGGGGGAACACCAGAATGAAGAAATGCCATTATGCACCTGCAGGAGCNNNNNNNNNNAAACATATCACTCCCATGTCAGTTTCTA<br>CAGCCGTAGTGGGCACTGCAACTCTCCAGTGGTTTGACTTTATCCCAATGGTGCACGCTTCCATTGTCTCTCGAGGCAAACGGATGCCAACA<br>AACCTTCACATACACATTCATTTA
2693823	TAACAAAATGACCATTGAATACAAATATGTCCTGATGCCAAACCGCTTGTGGCACTGCAGTGTCTATCTACAGGACCACTTTTTAGCTGAAG<br>GGTATGGCACAAAGAAAACCAGCAAGCATGCTGCAGCAGACGAGGCACTGAANNNNNNNNNNATGCTGGGGTCACCAAAGCAGTCCATG<br>TTCAGAAAGTTGGTGAGGCTGCCCGAGATTCCTCCAAGAAGAAGGATCTGAAGGACCTTGTTGTGTATGAGAACTCCATGAATCCTGTGTG<br>CACCCTGAATGACACAGCTCAGTTTA
2695650	TAAGCGACTTTGCTAGCATAGATTCAGCTGGGACCCTGAGTTCCAGTGATGAACCTGTGAGATTCAAAGCTGCAAATTCTCTGGAAATCAAG<br>CTGGAGAACTCCCAGATTGCAAATGTGATGCATGGGAGGAGGGAATGAAATCTCCAGCAAGCCTCAGCTTTTTACGGAGACGCAGCTGTGT<br>ATGTTACCTCTGGGAGATAATGCGGGCAGTGGTCCAAGTCAAGGGCTGGGAAGAATTTCCCTTGCAAAAACCTGCGAGAACGACCAGAAA<br>CTTA

2706132	TAAGTCTCCTTCACCAGTATTGCCTTGTTGAAGCAGTTACCACAATATTATTTCGATACCAGTATTCAATGATATGGCAGTTTATTACACAACCCTACTAGCTGGGAACTCCTTATTGTGTTCAGTACAGTCATACCTTAGTTGNNNNNNNNNNGGCTCCCAAACGCCGCAAACACAGAAGTGAGTGTTCCAGTTTGTGAATGTTTTTTGGAAGCCAAACATCTGGTGGGGCTTCTGCAGTTTTTGATTGGGTGAAGGAAACTCCTGCAGCCAATCGGAAGCCACACCTTGGAAGTTAATTGTGGAGACTACTTA
2709754	TAAAAGATTACAGCAGGACAAAAGGGCAGGGGAGGTCTCACAATAATGCTGGACCAGCCTAAATTCAACAGCGACATGCCAATATGGACTTTGCCAGGACTGTATGCCCTTGAATGAGCAACAGGGTCACCTGCCACCAATCCCNNNNNNNNNNTATCATTAGGGACTATGACAACTAGGATGGGGGCAAGGTTGCCACTACAGAAATGTGGAAGAACTGTGATGGGAGGCAGCTAAGAAGCAACAACAAGCTGTGGGTAGTGATGGACAGTCATCCACAGTAGCTCAAAAGTTTTTA
2811108	TAACTTAGTGTGAATGGAAGCAATTCTTCTGATGGCCTTGGACCATCATTTGATGAACTACCATCAACTGTTGGAGCTGCTCTCATCAGAAGGATCACTGTACATATGTTCATTAGTCCTAGATGTCTCATAACGTGTGATGCTNNNNNNNNNNTCATCATAGGGTTGAAAGTGGTGAGATGTTTGGATGCCAGTCAAATTGGTGACATTATTCCACCTTTCATCAATAAATGGTGTGTTTGACTTCACTTTTTTTTTTTTTTTTTTGAGTAACATACAAGATGTGCTTCTGTCAACTTTA
2811212	TAAACTTTTTATGTCCAACATCCTTTGCCCAATCTATCATAACAGATTTTACTTGTTCATCTTTCATATGCCACTCCAGCAATAAATTATACATTTTGGAAATATTCTTAGTCTTGGAACGCTTGGCAACTCTCACCTCGCTGGGCTNNNNNNNNNNAGAAGGATGGGGGGAAGGGAGGAGGTTGATGGTGTTTCTCACCGCCATGCCCCACATCAATGCAGCTGAATGCGGCTGGGAAGGGGAGCGCAGGGCGGGAAGGGGGAACATGGAGGTGGCACAAGCAGATTGCTGACCCCTTTA
2847708	TAAACAGCATGGGGGATAAAATCAAACCCTGAGGAACCCCACATTGAAGGCTTCACAGGGCTTCACAGCTCTCCCCAATCATCACCCTCTGGAAATGACCATCCAAGTAGGACTGGAACAACCACAAAGCTGGGCTCCCTATCCNNNNNNNNNNACTCCATTACATCAGCTCCTAAGGTGCAAGGATATGCACACCATTCCACACTAACTAGCTCCAAGGCAATCTACCTCTGCAGACTACTGCTCTGTCTTGGACAGTTTGAGCAATAGTATAGCCACTCCCCTTCCTGCTTTCTTA
2876301	TAAGCCCTGTATACCTGATGGAGTGTGTCTTCCCCCATCATTCAGCCTGGACACTGAGGTCCAGCGCTGTCAGGGGTTCACCCCAGAGTAGCAGAGTGATCAGTGAGGCTGAGCAGGAGGACAAGATAGAGGAAGAGGAGTGGGNNNNNNNNNNGGGGGCTCTTGGAGGCTCAGAGAGTCCCTGCATTGCCCAGGGAGAGAGAGGTGCCAGACCAGGGGGAGAGAGGGCGCCCCTTTAGCAGTTCTGGAACTGAGGGGGAGATCGTTCCCCCTTCACCCCAAGAGCGTAGGGAAGTTA
