# Supplementary material for: Sex-Linked Loci on the W Chromosome in the Multi-Ocellated Racerunner (Eremias multiocellata) Confirm Genetic Sex-Determination Stability in Lacertid Lizards
Source: Animals (Basel). 2023 Jul 3;13(13):2180. doi: 10.3390/ani13132180 (PMC10340011; doi:10.3390/ani13132180)
Supplement: Supplementary file 1 [file animals-13-02180-s001.zip › Table S5.pdf]

Table S5 The sex-linked loci of *Eremias multiocellata* and 15 (Z chromosome of *Eremias argus*) comparison results

Query id	Subject id	Identical(%)	s.start	s.end	e-value
CLocus_2509101	15	93.662	343298	343157	2.38E-53
CLocus_2613373	15	91.275	9768584	9768289	1.31E-115
CLocus_2619237	15	94.253	15646953	15647036	2.48E-28
CLocus_2623385	15	83.186	28365947	28365835	1.5E-20
CLocus_2627643	15	84.681	31510020	31510242	1.44E-55
CLocus_2630176	15	95.683	24802862	24802724	1.1E-56
CLocus_2632474	15	83.663	23336827	23336626	4.37E-51
CLocus_2634103	15	96.694	42671775	42671895	5.17E-50
CLocus_2638060	15	94.253	15647036	15646953	2.5E-28
CLocus_2644282	15	100	15571344	15571379	1.98E-09
CLocus_2651210	15	88.525	27525803	27525682	6.84E-34
CLocus_2652133	15	80.645	14607135	14606827	8.48E-63
CLocus_2652720	15	96.694	42671895	42671775	5.17E-50
CLocus_2652720	15	96.694	42671895	42671775	5.17E-50
CLocus_2657105	15	79.851	45901784	45901652	1.96E-14
CLocus_2657292	15	92.593	34282597	34282650	3.27E-12
CLocus_2693823	15	80.645	14606827	14607135	8.48E-63
CLocus_2706132	15	83.088	40940839	40940965	7.37E-24
CLocus_2785097	15	94.444	35605996	35605853	3.97E-56
CLocus_2811212	15	93.269	38886696	38886593	1.49E-35
CLocus_2847708	15	83.186	28365835	28365947	1.5E-20
CLocus_2876301	15	88.971	1438265	1438133	6.79E-39
CLocus_2876954	15	89.404	4177114	4176813	7.95E-108
