# Supplementary material for: Sex-Linked Loci on the W Chromosome in the Multi-Ocellated Racerunner (Eremias multiocellata) Confirm Genetic Sex-Determination Stability in Lacertid Lizards
Source: Animals (Basel). 2023 Jul 3;13(13):2180. doi: 10.3390/ani13132180 (PMC10340011; doi:10.3390/ani13132180)
Supplement: Supplementary file 1 [file animals-13-02180-s001.zip › Table S6.pdf]

Table S6 The sex-linked loci of *Eremias multiocellata* and NC _ 046330.1 (W chromosome of *Lacerta. agilis*) comparison results

**Query id**	**Subject id**	**Identical(%)**	**s.start**	**s.end**	**e-value**
CLocus_2847708	NC_046330.1	91.597	274596	274729	6.12E-41
CLocus_2623385	NC_046330.1	91.597	274932	274814	6.12E-41
CLocus_2648560	NC_046330.1	85.211	286461	286320	6.16E-36
CLocus_2811212	NC_046330.1	90.099	1762593	1762493	1.74E-31
CLocus_2642652	NC_046330.1	93.750	1786883	1787025	1.69E-56
CLocus_2656459	NC_046330.1	93.750	1787025	1786883	1.69E-56
CLocus_2629307	NC_046330.1	97.203	1794423	1794565	7.64E-65
CLocus_2709754	NC_046330.1	97.203	1794565	1794423	7.64E-65
CLocus_2651210	NC_046330.1	95.890	2332431	2332318	1.03E-33
CLocus_2657105	NC_046330.1	91.489	3942855	3943003	7.80E-50
